# Unification of optimal targeting methods in transcranial electrical stimulation

**DOI:** 10.1016/j.neuroimage.2019.116403

**Published:** 2019-12-17

**Authors:** Mariano Fernández-Corazza, Sergei Turovets, Carlos Horacio Muravchik

**Affiliations:** a LEICI Instituto de Investigaciones en Electrónica, Control y Procesamiento de Señales, Universidad Nacional de La Plata, CONICET, Argentina; b NeuroInformatics Center, University of Oregon, Eugene, OR, USA; c Comisión de Investigaciones Científicas, CICPBA, Provincia de Buenos Aires, Argentina

**Keywords:** Transcranial electrical stimulation (TES), Transcranial direct current stimulation (tDCS), Optimal electrical stimulation, Reciprocity theorem, Least squares

## Abstract

One of the major questions in high-density transcranial electrical stimulation (TES) is: given a region of interest (ROI) and electric current limits for safety, how much current should be delivered by each electrode for optimal targeting of the ROI? Several solutions, apparently unrelated, have been independently proposed depending on how “optimality” is defined and on how this optimization problem is stated mathematically. The least squares (LS), weighted LS (WLS), or reciprocity-based approaches are the simplest ones and have closed-form solutions. An extended optimization problem can be stated as follows: maximize the directional intensity at the ROI, limit the electric fields at the non–ROI, and constrain total injected current and current per electrode for safety. This problem requires iterative convex or linear optimization solvers. We theoretically prove in this work that the LS, WLS and reciprocity-based closed-form solutions are specific solutions to the extended directional maximization optimization problem. Moreover, the LS/WLS and reciprocity-based solutions are the two extreme cases of the intensity-focality trade-off, emerging under variation of a unique parameter of the extended directional maximization problem, the imposed constraint to the electric fields at the non–ROI. We validate and illustrate these findings with simulations on an atlas head model. The unified approach we present here allows a better understanding of the nature of the TES optimization problem and helps in the development of advanced and more effective targeting strategies.

## 1. Introduction

Transcranial electrical stimulation (TES) is an emerging therapy for the treatment of neuropsychiatric conditions such as clinical depression (Kalu et al., 2012), Parkinson’s disease (Boggio et al., 2006), anxiety and chronic pain (Mori et al., 2010). Research has also shown that TES can be a valuable therapeutic tool in epilepsy (Yook et al., 2011), stroke rehabilitation (Schlaug et al., 2008), and other neurological and psychiatric disorders (Brunoni et al., 2013). It has also been extensively studied in the context of enhancing cognitive skills such as memory and learning (Nitsche et al., 2003; Berryhill and Jones, 2012). This technique may eventually become an alternative for psychoactive drugs, as it can be more selective than drugs by targeting specific regions of interest in the brain with minimal adverse side effects. Even without producing direct neuronal firing, TES application is capable of modifying cortical excitability (Priori et al., 1998; Nitsche and Paulus, 2000) as well as brain rhythms and networks (Priori, 2003; Lang et al., 2005), and this is why the method is also named Transcranial Electrical Neuromodulation (TEN). Because the goal is to stimulate the brain, TES is also termed Transcranial Brain Stimulation (TBS). If direct or alternating currents are used, TES is termed transcranial direct current stimulation (tDCS) or transcranial alternating current stimulation (tACS), respectively. Despite recent advances, there are ongoing debates on the clinical effectiveness of TES (Horvath et al., 2014, 2015; Antal et al., 2015) addressing many issues still to be resolved, in particular, substantial inter-subject response variability (Batsikadze et al., 2013; Wiethoff et al., 2014). The general idea is that optimal targeting protocols and the use of subject-specific accurate head models might enhance rigor and reproducibility in TES (Bikson et al., 2018).

In TES, electric currents are applied to two or more electrodes placed on the scalp. If the number of electrodes is larger than 2, it is called multi-electrode TES. If it is even larger, being for instance 32, 64, 128 or 256 like typically arranged in high channel count electroencephalography (EEG), it is known as high-density TES. A list of electric current levels applied to the head at each electrode is known as a current injection pattern, which produces an electric field (or current density) map on the brain that can be considered as the actual dose in TES. The computation of this map is based on the electromagnetic physical laws and known as the TES forward problem (FP). The TES FP equations are typically solved numerically using the finite element method (FEM) (Datta et al., 2013), boundary element method (BEM) (Goncalves et al., 2003) or finite difference method (FDM) (Turovets et al., 2014).

The inverse problem (IP) goal in high-density TES is to determine current injection patterns for optimally targeting a specific region of interest (ROI) within the brain. When solving the TES inverse problem, one should address a trade-off between maximizing the electric field at the ROI and limiting or minimizing it at the non–ROI while constraining the values of the applied currents to meet safety standards. The two common limits for the electric currents are: total injected current (or “fixed budget”) and maximum current per electrode. Depending on the optimality criteria, several schemes have been proposed leading to different optimal solutions.

**Least Squares (LS) and Weighted-LS (WLS)** are the simplest and most typical optimization methods. The LS solution derives from minimizing a second-order error between the resulting and the desired electric field (or current density) profiles at a specific domain of interest Ω. Typically, these profiles are non-zero at the ROI part of Ω (*Ω*_ROI_) and zero at the non–ROI part of Ω (*Ω*_non–ROI_). Domain Ω can be any volume that includes the ROI and the regions where stimulation should be minimized or limited, such as the brain (e.g. Dmochowski et al., 2011; Guler et al., 2016a), the gray matter, or the full head (Fernández-Corazza et al., 2016). WLS is similar to LS with the addition of a weight matrix that, for instance, can control the intensity-focality trade-off (Dmochowski et al., 2011) or incorporate additional *a-priori* knowledge (Ruffini et al., 2014). If no additional current injection limits are imposed, the LS or WLS solutions follow a well-known closed-form (Dmochowski et al., 2011; Fernández-Corazza et al., 2016; Salman et al., 2016). One option to account for the total current budget constraint without the need of iterative solvers is to apply a scaling factor to the closed-form LS/WLS solution (as in Fernández-Corazza et al., 2016; Dmochowski et al., 2017) here designated as “optimally scaled WLS”. Another option is to include the total and per electrode current limits explicitly, and solve the problem using an iterative optimization algorithm such as LASSO (Dmochowski et al., 2011) or MATLAB convex optimization (Dmochowski et al., 2011). Limiting the number of active electrodes was also proposed and solved using genetic algorithms (Ruffini et al., 2014; Otal et al., 2016) and the branch and bound algorithm (Guler et al., 2016b). The LS based optimization was also earlier formulated in the context of multichannel Transcranial Magnetic Stimulation (TMS) (Ilmoniemi et al., 1999).

**Constrained directional maximization** of the electric field (or current density) intensity at the ROI along a predefined and desired orientation is another optimization approach. It can be numerically solved with convex optimization algorithms such as those included in the “CVX: Matlab Software for Disciplined Convex Programming” package (Grant and Boyd, 2014). In this approach, the functional to be maximized is linear with respect to the unknown current injection pattern, thus it requires some limiting constraints to get finite solutions. The simplest constraint is to consider only the total current limit (Eq. 17 in Dmochowski et al., 2011). Later, Guler et al., (2016a, 2018) and Wagner et al. (2016a) included upper bounds for the undesired electric field at *Ω*_non–ROI_ and per-electrode current limits as additional constraints, constituting an extended directional maximization problem. The non–ROI constraints can be either the global integral of the electric field energy (Guler et al., 2016a), or the electric field maximum intensity at each point in the space (Wagner et al., 2016a) or non–ROI subdomain (Guler et al., 2018).

**Reciprocity-based** optimization solutions are based on the reciprocity theorem in EEG (Rush and Driscoll, 1969; Malmivuo and Plonsey, 1995). In this approach, optimal stimulation patterns are derived from the EEG forward projection to the scalp of source dipoles artificially placed at the ROI and oriented in the direction of interest (Dutta and Dutta, 2013; Ruffini, 2015; Cancelli et al., 2016; Fernández-Corazza et al., 2016; Salman et al., 2016). Here, “EEG forward projection” refers to the electric potential on the scalp produced by the neuronal sources (typically modelled as electrical dipoles), i.e. the solution of the EEG FP. One reciprocity approach is to concentrate the electric current sources and sinks as close as possible to the “poles” of the EEG forward projection (Fernández-Corazza et al., 2015, 2016; Guhathakurta and Dutta, 2016). These EEG forward projection poles denote the two points on the scalp with the largest electric potential difference. In our previous work, we mathematically proved that this strategy maximizes the directional electric field at the ROI given a fixed current injection budget (Fernández-Corazza et al., 2016). Another approach is setting the current injection pattern proportionally to the EEG forward projection, either directly or after applying a Laplacian filter (Dutta and Dutta, 2013; Cancelli et al., 2016), though we found that its performance was not better in any of the tested metrics compared to other approaches (Fernandez-Corazza et al., 2017). As the reciprocity-based solutions are not iterative, they can be also considered “closed-form” solutions.

In this work, we link these three apparently unrelated optimization methods and some of their variants resulting in a unified formulation that couples together most optimization schemes described so far (see Table 1 for a list of covered methods). As far as we know, the links we present here have not been fully noticed previously and they are a major novelty of this work. In Section 2, we briefly describe the computational methods for the TES FP. In Section 3.1, we describe the details of the constrained directional maximization approaches. Then, we theoretically link this iterative approach first to LS and WLS solutions (in Section 3.2), and second, to reciprocity-based solutions (in Section 3.3). In Section 4, we illustrate these links with two sets of simulations on a virtual head model. With the first set, we show how the directional maximization iterative solutions evolve from the WLS to the reciprocity closed-form solutions when varying the imposed bound to the energy integral over *Ω*_non–ROI_. The second set is like the first set, but with electric field intensity limits at each point in *Ω*_non–ROI_ instead of a unique global restriction for the integral of the electric field energy over *Ω*_non–ROI_. The way we present the different optimal solutions in a unified formulation is also a novelty of this work. It offers a clear visualization and quantification of the well-known intensity versus focality trade-off to select the most adequate targeting strategies for each practical case.

## 2. TES forward problem

Due to the low frequencies involved, the FP is governed by the quasistatic Maxwell equations. It is described by the Poisson equation for the electric potential $\text{ψ}(\vec{x})$ in the head volume with Neumann boundary conditions (Frank, 1952; Jackson, 1975). Boundary conditions differ in approximation of pointwise or distributed electrodes. In the latter case, they are modelled using the complete electrode model (CEM) (Hyvönen, 2004). The FP is typically solved using the Finite Element Method (FEM) (Silvester and Ferrari, 1994; Kwon and Bang, 2000), where the whole head is meshed into *N*_*H*_ elements, usually tetrahedrons, and *P* nodes. Details of the FEM FP formulation in TES can be found for example in (Vauhkonen et al., 1999; Windhoff et al., 2013; Ruffini et al., 2014; Laakso et al., 2016). The TES forward problem is equivalent to the Electrical Impedance Tomography (EIT) FP, and thus, EIT literature also details the same FEM formulation (Lionheart et al., 2004; Abascal et al., 2008; Wang et al., 2009; Fernández-Corazza et al., 2013).

The FEM converts the FP formulation into a linear system of equations **Kv** = **u**, where **K** is the *stiffness matrix* and accounts for the geometry, bulk conductivity or a conductivity map of each tissue, and electrode contact impedances (if using CEM); **v** is the vector of unknown electric potentials at each mesh node of the head and at the electrodes, and **u** is a vector accounting for the electric sources and sinks (in TES, the applied currents or, equivalently, the current injection pattern). Once the system of linear equations above is solved for **v**, for instance, using the iterative preconditioned conjugate gradient (Barrett et al., 1994) or the biconjugate stabilized gradient (van der Vorst, 1992) solvers, the electric field $\vec{E}(\vec{x})$ can be easily computed at each element by: $\vec{E}(\vec{x})=-\vec{\nabla }(\text{ψ}(\vec{x})),\;\text{where}\;\vec{\nabla }$ is the gradient operator.

## 3. Unification of optimization approaches

Table 1 summarizes different optimization methods covered by this unified approach. The first five rows correspond to variants of the LS methods (pale pink background), sixth and seventh rows are variants of the constrained directional maximization methods (white background), and the last two rows correspond to reciprocity-based methods (pale blue background). We describe the constrained directional maximization method in Section 3.1 and we theoretically link it to the WLS and reciprocity-based solution methods in Sections 3.2 and 3.3, respectively.

Other less common optimization approaches that are not considered in this work have been proposed in the literature. One of them is beamforming or Linearly Constrained Minimum Variance (LCMV) (Dmochowski et al., 2011; Fernández-Corazza et al., 2016). This approach imposes that the electric field at the ROI or target is totally collinear with a desired targeting orientation. Similarly to LS or WLS, it has a closed-form solution when no current limits are considered. Another approach maximizes the modulus of the electric field at the ROI instead of the directional intensity (Sadleir et al., 2012). This problem, although it has great interest for multiple applications, is much more difficult to solve as it is nonconvex and nonlinear. The authors attempted to solve it using the interior point optimization algorithm, but they concluded that there is no guarantee that the solution they found is a global optimum due to the complex nature of this optimization problem (Sadleir et al., 2012). Although Ruffini et al. (2014) used a formulation of the WLS problem, their treatment additionally imposes a limit on the number of active electrodes that we do not consider here. Finally, Guler et al. (2016b) proposed reducing the number of active electrodes and solved it with the branch and bound algorithm.

### 3.1. Constrained directional maximization approaches

The constrained maximization approaches consider the maximization of the integral over *Ω*_ROI_ of the local electric field $\vec{E}(\vec{x})$ (or current density) projection onto a desired unitless orientation $\vec{d}(\vec{x})$. The three typical constraints are: (i) upper limits for the electric _fi_eld in *Ω*_non–ROI_, (ii) a total current limit or “budget”, and (iii) current limits per electrode. For constraint (i), an option is to constrain the integral of the electric field (or current density) energy over *Ω*_non–ROI_ by an arbitrary scalar α_I_, where subindex I stands for “integral” (Guler et al., 2016a). Another option is to impose a set of constraints: upper bounds $\alpha_{\text{E}}(\vec{x})$ for the electric field at each *Ω*_non–ROI_ point or subdomain, as proposed in Wagner et al. (2016a) and Guler et al., (2018), where sub-index E stands for “elementwise”. If the upper bound is equal for all *Ω*_non–ROI_ points or subdomains, this latter approach means constraining the maximum intensity at *Ω*_non–ROI_. The mathematical formulation considering both alternatives can be stated as follows:

(1) $$\hat{i}=\underset{i}{\mathrm{argmax}}\left(\int_{\Omega_{\text{ROI}}}\vec{E}(\vec{x})\cdot \vec{d}(\vec{x})d\vec{x}\right),\;subject\;to\;(\text{i})\;\{\begin{array}{l} (\text{i.a})\;\int_{\Omega_{\text{non–ROI}}}\|\vec{E}(\vec{x}){\|}_{2}^{2}d\vec{x}\leq \alpha_{\text{I}}\\ (\text{i.b})\|\vec{E}(\vec{x}){\|}_{2}\leq \alpha_{\text{E}}(\vec{x}),\;\forall \vec{x}\in \Omega_{\text{non–ROI}} \end{array}\;\text{or}\;(\text{ii})\;\|\tilde{i}{\|}_{1}\leq 2i_{\max}\;(\text{iii}){\tilde{i}}_{\min}≼\tilde{i}≼{\tilde{i}}_{\max}$$

where **i** is the unknown (*L* − 1) × 1 current injection pattern (where *L* is the number of electrodes); *i*_*max*_ is the maximum total current intensity scalar; $\tilde{i}$ is the expanded current injection pattern vector of size *L* × 1 that considers all electrodes; ${\tilde{i}}_{\min}\;\text{and}\;{\tilde{i}}_{\max}$ are the *L* × 1 minimum and maximum limits per electrode respectively; symbol ≼ means “≤” but elementwise; and ‖·‖_1_ is the $l_{1}$-norm (sum of absolute values of all vector components). For *L* electrodes, there are (*L* − 1) independent current injection electrodes (pattern **i**), as the remaining electrode (the last element of expanded pattern $\tilde{i}$) is the sum of all other currents such that total injected current is zero, i.e., Kirchhoff’s Law (see also section 2.2 in Guler et al. (2016a) and Eq. (10) constraint in Dmochowski et al. (2011):

(2) $$\{\begin{array}{ll} {\tilde{i}}_{j}=i_{j}, & \forall j=1,\ldots ,L-1\\ {\tilde{i}}_{L}=-\sum_{l=1}^{L-1}i_{l} & \; \end{array}$$

Note that $\tilde{i}=H\cdot i,$ with **H** being the *L* × (*L* − 1) matrix $\left[\begin{array}{c} I_{L-1}\\ -1-1\ldots -1 \end{array}\right],$ where **I**_*L* − 1_ is the *L* − 1 identity matrix.

Assuming *N* total brain mesh elements, we define **T** as the TES 3*N* × (L − 1) *transfer matrix* where each column “*l*” is the TES FP solution (i.e., the x, y and z components of the electric field) computed as described in Section 2, caused by a current injection pattern that consists of injecting the electric current at electrode *l* with last electrode *L* being the sink (or reference). Note that for *L* electrodes, there are *L* − 1 independent current injection patterns. All other patterns can be generated from this *basis* by superposition. Other bases can be used such as injecting the electric current at electrode *l* and assuming all other *L* − 1 electrodes as sinks (as used in Fernández-Corazza et al. (2016)). Note that **T** can be reduced to cover only the gray matter or expanded to cover other head regions of interest to stimulate or to avoid stimulation such as the optic nerves, the eyes, facial muscles, etc.

The constrained directional maximization problem in Eq. (1) can be re-stated in a discrete form as:

(3) $$\begin{array}{l} \hat{i}=\underset{i}{\mathrm{argmax}}\left(d^{\text{T}}\Gamma Ti\right),\;s.t.\\ (\text{i})\;\{\begin{array}{l} \;(\text{i.a})\;i^{\text{T}}T^{\text{T}}\Gamma_{\text{non–ROI}}Ti\leq \alpha_{\text{I}}\\ (\text{i.b})\;{\left\|T_{\text{n}}i\right\|}_{2}\leq \alpha_{\text{E}}[n],\forall n\in \Omega_{\text{non–ROI}} \end{array}\;\text{or}\\ (\text{ii})\;\|\tilde{i}{\|}_{1}\leq 2i_{\max}\\ (\text{iii})\;{\tilde{\text{i}}}_{\text{min}}≼\tilde{\text{i}}≼{\tilde{\text{i}}}_{\text{max}} \end{array}$$

where **d** is the 3*N* × 1 vector representing an *N* point discretization of the directional vector field $\vec{d}(\vec{x})$ for desired orientation of the stimulation field in the brain, with non-zero values at *Ω*_ROI_ and zero values at *Ω*_non–ROI_. The non-zero values of **d** are typically unitary vectors oriented perpendicularly to the cortical surface, but they can be, in general, oriented in any direction and have different strengths. **T**_*n*_ is the 3 × (*L* − 1) transfer matrix of each non–ROI element *n*.

Volume matrices **Γ** and **Γ**_non–ROI_ stem from the integration operations in Eq. (1). **Γ** is a diagonal 3*N* × 3*N* matrix where each element of the diagonal is the volume of each mesh element.^1^ If **Γ** is equal to the identity matrix, it means that the sum across the mesh elements is used instead of the volume integral (as in Eq. (17) in Dmochowski et al. (2011)). In the *Ω*_non–ROI_ electric field energy constraint of Eq. (1.i), the integral is taken over the non–ROI, hence, **Γ**_non–ROI_ is obtained from **Γ** by setting the diagonal elements corresponding to the ROI to zero. Note that the *Ω*_ROI_ is typically much smaller than *Ω*_non–ROI_, thus **Γ**_non–ROI_ ≈ **Γ** with almost any matrix norm. This approximation can also be interpreted as integrating the constraint of Eq. (1.i) over the whole domain of interest *Ω*, and not just over *Ω*_non–ROI_^2^.

Optimization problem in Eq. (3) is a convex optimization problem (Boyd and Vandenberghe, 2004), where the objective function is a linear function, constraints in Eqs. (3.i.a) and (3.i.b) are quadratic, and constraints in Eqs. (3.ii) and (3.iii) can be formulated as linear inequalities (more details can be found in Appendices A and B). Thus, this problem can be categorized as a quadratically constrained linear program (QCLP).

Matrix **T** in Eq. (3) is the *electric field* transfer matrix as explained before. Alternatively, one can consider the matrix product **ΣT** as a *current density* transfer matrix **T**’ instead of **T** in Eq. (3). In such case, the conductivity matrix **Σ** is a 3*N* × 3*N* symmetric block diagonal matrix where each 3 × 3 block of the diagonal is the conductivity tensor of the mesh element *n*. If piecewise isotropic media is assumed, **Σ** is a diagonal matrix, and moreover, if only one homogeneous and isotropic conductivity value σ_*B*_ is assumed for the whole region covered by **T**, matrix **Σ** can be replaced by the scalar *σ*_*B*_.

### 3.2. Link between constrained directional maximization and LS approaches

We first assume in Eq. (3) that the integral over *Ω*_non–ROI_ of the electric field energy constraint (3.i.a) dominates (i.e., α_I_ is low, and thus the total injected current constraint (ii) can be neglected), the electric current per electrode bounds (iii) are ${\tilde{i}}_{\max}=i_{\max}1\;\text{and}\;{\tilde{i}}_{\min}=-i_{\max}1\;(\text{where}\;1$ is a vector with all ones), i.e., it is allowed that just one electrode pair can inject the maximum allowed current, and that **Γ**_non–ROI_ ≈ **Γ** holds. With these assumptions, Eq. (3) is reduced to:

(4) $$\hat{i}=\underset{i}{\mathrm{argmax}}\left(d^{\text{T}}\Gamma Ti\right),\;\text{s.t.}\;i^{\text{T}}T^{\text{T}}\Gamma Ti\leq {\text{α}}_{\text{I}}$$

The constrained maximizing intensity problem in Eq. (4) belongs to a class of QCLP and, the solution, if not infinity or minus infinity, lies at the boundary, i.e. at **i**^T^**T**^T^**ΓTi** = α_I_ (note the “=” instead of the “≤” sign) (Boyd and Vandenberghe, 2004). In Appendix A we prove, analytically solving the Karush-Kuhn-Tucker (KKT) conditions,^3^ that the solution to Eq. (4) has the form:

(5) $$\hat{i}={\left(T^{\text{T}}\Gamma T\right)}^{-1}T^{\text{T}}\Gamma kd,\;\text{with}\;k\left(\alpha_{\text{I}}\right)=\sqrt{\alpha_{\text{I}}/d^{\text{T}}\Gamma T{\left(T^{\text{T}}\Gamma T\right)}^{-1}T^{\text{T}}\Gamma d}$$

where *k*(α_I_) is a scaling constant expressed in [*V*/*m*]. Solution in Eq. (5) is also the known analytical solution of a typical WLS problem (an unconstrained quadratic problem) of the form:

(6) $$\hat{i}=\underset{i}{\mathrm{argmin}}\left\{{\left(kd-Ti\right)}^{\text{T}}\Gamma (kd-Ti)\right\}$$

Note that in Eq. (6), *k***d** plays the role of a desired electric field in the WLS formulation.^4^ On one hand, given an arbitrarily imposed α_I_ value in Eq. (4), the formulation in Eq. (5) gives the corresponding value of *k* and the closed-form solution to problem (4). On the other hand, if a desired electric field **f** = *k***d** (in V/m) is imposed in the WLS formulation of Eq (6), one can always assume *k* = 1V/m. Then, **d** is equivalent to **f** but unitless, and the value of α_I_ that makes Eqs. (4) and (6) to be equivalent can be derived directly from Eq. (5).

If the approximation **Γ**_non–ROI_ ≈ **Γ** is not considered, the solution to Eq. (4) becomes $\hat{i}={\left(T^{\text{T}}\Gamma_{\text{non}-\text{ROI}}T\right)}^{-1}T^{\text{T}}\Gamma dk,$ with $k=\sqrt{{\text{α}}_{\text{I}}/{\text{d}}^{\text{T}}\Gamma T{\left(T^{\text{T}}\Gamma_{\text{non}-\text{ROI}}T\right)}^{-1}T^{\text{T}}\Gamma d}.$ This is not exactly a WLS solution because **Γ**_non–ROI_ ≠ **Γ**, but it is extremely similar if *Ω*_ROI_ is much smaller than *Ω*_non–ROI_, and still has a closed-form solution. If **Γ** is the identity matrix, the equivalence between Eqs. (4) and (6) still holds, and the solution has the LS form: $\hat{i}={\left(T^{\text{T}}T\right)}^{-1}T^{\text{T}}dk,\;\text{with}\;k=\sqrt{\alpha_{\text{I}}/d^{\text{T}}T{\left(T^{\text{T}}T\right)}^{-1}T^{\text{T}}d}.$

Overall, if the integral over *Ω*_non–ROI_ in Eq. (3.i.a) is low enough such that the solution to Eq. (3) requires the injection of less current than the total maximum allowed, the shape of the current injection pattern maintains the LS/WLS closed-form regardless of the value of α_I_, and α_I_ only plays the role of a scaling factor. **Thus, the LS/WLS solutions belong to a limit case of the constrained directional maximization problem of Eq. (3), the one for low α values**.

#### 3.2.1. Links with additional constraints

In addition, we also show in Appendix A that the following two problems (with total budget constraints added in comparison to Eqs. (4) and (6)):

(7a) $$\hat{i}=\underset{i}{\mathrm{argmax}}\left(d^{\text{T}}\Gamma Ti\right),\;s.t.\;i^{\text{T}}T^{\text{T}}\Gamma Ti\leq \alpha_{\text{I}}’\;and\;\|\tilde{i}{\|}_{1}\leq 2i_{max}$$

(7b) $$\hat{i}=\underset{i}{\mathrm{argmin}}\left\{{\left(kd-Ti\right)}^{\text{T}}\Gamma (kd-Ti)\right\},\;s.t.\;\|\tilde{i}{\|}_{1}\leq 2i_{max}$$

also have the same KKT conditions for ${\text{α}}_{1}^{\prime }={\hat{i}}^{\text{T}}T^{\text{T}}\Gamma T\hat{i},\;\text{with}\;\hat{i}$ being the optimal solution of the constrained WLS problem in Eq. (7b) assuming *k* = 1V/m. The difference between this equivalence and the equivalence previously shown in Eqs. (4) and (6) is that now the KKT conditions do not have a closed-form solution and an iterative solver such as one of those provided by the CVX Matlab package is required. In the proof of Appendix A, the $l_{1}$-norm constraint in Eqs. (7a) and (7b) is converted into a set of linear constraints. Thus, Eq. (7a) belongs to a class of QCLP while Eq. (7b) is a Linearly Constrained Quadratic Program (LCQP). Note that Eq. (7b) is the same as the problem of the fourth row in Table 1 solved using the LASSO algorithm in (Dmochowski et al., 2011).

Moreover, one can further complicate Eqs. (7a) and (7b) by adding the current per electrode constraints. Again, the following two problems (also a QCLP, and an LCQP):

(8a) $$\hat{i}=\underset{i}{\mathrm{argmax}}\left(d^{\text{T}}\Gamma Ti\right),\;s.t.\;i^{\text{T}}T^{\text{T}}\Gamma \mathrm{Ti}\leq \alpha_{\text{I}}”,\;\|\tilde{i}{\|}_{1}\leq 2i_{max}\;and\;{\tilde{i}}_{\text{min}}≼\tilde{i}≼{\tilde{i}}_{\max}$$

(8b) $$\hat{i}=\underset{i}{\mathrm{argmin}}\left\{{\left(kd-Ti\right)}^{\text{T}}\Gamma (kd-Ti)\right\},\;s.t.\;\|\tilde{i}{\|}_{1}\leq 2i_{max}\;and\;{\tilde{i}}_{\min}≼\tilde{i}≼{\tilde{i}}_{\max}$$

have the same KKT conditions for ${\text{α}}_{\text{I}}^{”}={\hat{i}}^{\text{T}}T^{\text{T}}\Gamma T\hat{i},\;\text{now with}\;\hat{i}$ being the optimal solution of the constrained WLS problem in Eq. (8b) (assuming *k* = 1V/m). This is expected because if the KKT conditions for Eqs. (7a) and (7b) are equivalent, then adding the same additional set of constraints modifies the KKT for both problems in the same way.

### 3.3. Link between constrained maximizing intensity and reciprocity

In this section we show that when omitting the constraint (3.i) in Eq. (3), the iterative solution is equivalent to the closed-form reciprocity-based solution.

The reciprocity theorem coupling TES and EEG for one dipole and one injection pair states that given a dipole at position $\vec{x}$ with dipolar moment $\vec{m}\;[\text{A}.\text{m}],$ the electric potential (Φ) difference between any points *a* and *b* on the scalp can be computed as the dot product:

(9) $$\Phi (a)-\Phi (b)=\frac{\vec{m}\cdot \vec{\nabla }\psi_{\text{ab}}(\vec{x})}{I_{ab}},$$

where ${\text{ψ}}_{\text{ab}}(\vec{x})$ is the resulting potential at location $\vec{x}$ when an electric current *I_ab_* is injected at the arbitrary points *a* and *b* (Malmivuo and Plonsey, 1995; Rush and Driscoll, 1969). In our previous work we showed that, as a direct consequence of Eq. (9), if the poles of the EEG forward projection are used for two-electrode stimulation, the dot product of the electric field and the desired orientation is maximized (Fernández-Corazza et al., 2016). Mathematically,

(10) $$A,B=\underset{a,b}{\mathrm{argmax}}\{\Phi (a)-\Phi (b)\}=\underset{a,b}{\mathrm{argmax}}\left\{\frac{\vec{\nabla }{\text{ψ}}_{ab}(\vec{x})}{I_{ab}}\cdot \vec{m}\right\}\;\Leftrightarrow \vec{\nabla }{\text{ψ}}_{AB}(\vec{x})\cdot \vec{m}\;\text{is maximal}.$$

In this work, we go a step further and explicitly link the same reciprocity-based approaches of our previous work with the directional maximization problem in Eq. (3). For this link to be valid, we assume that α_I_ or α_E_ is large enough such that the total current limit constraint in Eq. (3.ii) dominates over the *Ω*_non–ROI_ energy limit in Eq. (3.i). Note that this assumption results in a similar problem to the simpler maximizing intensity approach of Eq. (17) in Dmochowski et al. (2011), formulated therein for a pointwise ROI. Also note that this case is opposite to the extreme case considered in the previous Section 3.2, where the *Ω*_non–ROI_ energy limit in Eq. (3.i) dominates over the total current limit constraint in Eq. (3.ii).

Eq. (9) can be generalized for multiple dipoles and multiple injection pairs, implying that the elements of TES transfer matrix **T** and EEG lead field matrix **L** are related by transposition: **T** = **L**^T^ (both in [V/(Am)]). Each column of **L** corresponds to the electric potential at *L* − 1 electrodes (assuming electrode *L* as the reference) due to a unit dipole at a canonical orientation located at each cortical (or brain) element. Thus, matrix **L** has size *L* − 1 × 3*N*. The fact that **T** = **L**^T^ derives from the reciprocity principle in Eq. (9) is well known and proven in the literature (Weinstein et al., 2000; Wolters et al., 2004; Hallez et al., 2005; Malony et al., 2011; Wagner et al., 2016b), see also more recent discussions in Dmochowski et al. (2017) and Salman et al. (2016). Then, the linear functional to be maximized in Eq. (3) can be written as:

(11) $$\hat{i}=\underset{i}{\mathrm{argmax}}\left(d^{\text{T}}\Gamma Ti\right)=\underset{i}{\mathrm{argmax}}\left(sd^{\text{T}}\Gamma L^{\text{T}}i\right)=\underset{i}{\mathrm{argmax}}\left(\Phi_{\Gamma }^{\text{T}}i\right)$$

Φ_Γ_ = **LΓd***s* is a synthetic potential at the electrodes generated by the EEG dipolar source field *s***d**^T^**Γ**, which is shaped by the desired orientation vector field **d** with the magnitude given by an arbitrary constant and positive dipole source density *s* (in [Am/m^3^]). Note that the effect of **Γ** is just weighting the strength of each dipolar source according to the volume of the containing element.

Now, Eq. (3) is reduced to the $l_{1}$-constrained linear optimization problem

(12) $$\hat{i}=\underset{i}{\mathrm{argmax}}\left(\Phi_{\Gamma }^{\text{T}}i\right)\;s.t.$$

$$\|\tilde{i}{\|}_{1}\leq 2i_{max}$$

Note that $\Phi_{\Gamma }^{\text{T}}i=\sum_{l=1}^{L-1}\varphi_{l}i_{l},\;\text{where}\;\varphi_{l}$ is the EEG potential at the *l*^th^ electrode. As this problem has a $l_{1}$-norm constraint, the most typical approach for solving it until now has been using iterative solvers. We can now prove that the solution to Eq. (12) is:

(13) $$\hat{\tilde{i}}=i_{max}e_{l_{max}}-i_{\max}e_{l_{min}}$$

where **e**_*l*_ is a zero *L* × 1 vector with a “1” at element “*l*”,^5^ *l*_max_ is the electrode with maximum Φ_Γ_ and *l*_*min*_ is the electrode with minimum Φ_Γ_.

Since the functional to maximize in Eq. (12) is linear, the fundamental theorem of linear programming states that the solution to Eq. (12) belongs to the boundary, i.e., when $\|\tilde{i}{\|}_{1}=2i_{max}$ (Luenberger and Ye, 2008). Moreover, the same theorem states that if the feasible domain is a bounded polyhedron (as the $l_{1}$-norm defines), the solution occurs at a domain’s corner. The next step is to prove that the corners in the feasible domain of Eq. (12) only have two active electrodes. In Appendix B, we depict the feasible domains for two and three electrodes (2D and 3D geometrical representations) showing that, effectively, their vertices correspond to only two active electrodes. Then, we extend the proof for larger dimensions. Finally, among all possible pairwise solutions, it is obvious that picking the two electrodes with maximum Φ_Γ_ difference also maximizes Φ_Γ_^T^**i**. **Thus, the reciprocity-based optimization approach is the solution that belongs to another limit case of the constrained directional maximization problem in Eq. (3), the one with high α values**.

#### 3.3.1. Considering maximum current per electrode limit

If we include maximum current per electrode limit constraints ${\tilde{i}}_{\min}≼\tilde{i}≼{\tilde{i}}_{\max},$ closed-form solutions like Eq. (13) can be derived using a similar reasoning as described above. The details can be found in Appendix B. If ${\tilde{i}}_{\min}\;\text{or}\;{\tilde{i}}_{\max}$ are the same for all electrodes, the resulting solution has groups of neighboring electrodes injecting the same amount of current, imitating TES “patches”. For instance, suppose that we set ${\tilde{i}}_{\max}=\left(i_{\max}/2\right)1\;\text{and}\;{\tilde{i}}_{\min}=\left(-i_{\max}/20\right)1.$ This means that the solution will have at least two sources to reach the upper current limit and maximally twenty sinks to fulfill Kirchhoff’s law. To maximize Φ^T^**i**, the two electrodes with maximum Φ with respect to the reference electrode *L* should be selected as sources to inject *i*_*max*_/2 and the 20 electrodes with minimum Φ with respect to *L* should be selected as sinks to inject – *i*_*max*_/20. Similarly, it is possible to obtain the “opposite”, “one source-all sinks”, and “10 sources-30 sinks” schemes suggested in Fernández-Corazza et al. (2016) by solving Eq. (3) with corresponding maximum current per electrode constraints imposed by Eq. (3.iii).^6^

## 4. Simulations

In this section we illustrate our analytical findings with simulations using a head model based on the ICBM-152 symmetric atlas (Mazziotta et al., 2001). The unified visualization scheme we use here to present the results can help a potential TES planner to determine the best stimulation strategy according to the experimental criteria and specific needs.

### 4.1. Simulation framework

We used a head model with four tissues: brain, CSF, skull and scalp based on the ICBM-152 atlas, which is an average of 152 individual heads (Mazziotta et al., 2001). Base-line triangular surfaces were obtained from the SPM8 MATLAB package (Friston, 2007) and further refinement, smoothing and tetrahedral meshing was performed using the Iso2mesh MATLAB package (Fang and Boas, 2009). The final tetrahedral mesh had ~1 million elements and ~150k nodes. We assumed homogeneous and isotropic conductivities for each tissue assigning literature values: 0.3, 0.006, 1.79, and 0.33 S/m for the scalp, skull, CSF, and brain, respectively (Gabriel et al., 1996; Baumann et al., 1997; Fernandez-Corazza et al., 2018). The model is completed with 64 pointwise electrodes placed following a subset of the standard 10–10 EEG electrode coordinates. All algorithms can be applied to more complex models with different conductivity values and number of electrodes, as theoretical findings described in previous sections are model-independent.

We selected a part of the M1 cortical region of ~1.4 cm^3^ as *Ω*_ROI_ or target. For each tetrahedral element of the *Ω*_ROI_, its centroid was projected to the closest triangular element of the external brain surface and the normal to the cortex vectors of these surface triangles were computed. A vector representing an average orientation of the ROI was defined as a weighted by element volume average of these surface triangle normal vectors. Then, this unique orientation was replicated in each *Ω*_ROI_ element to form the target vector **d**. Note that any other orientation, even arbitrary, can be used instead. The transfer matrix **T** was obtained as described in Section 2 using our MATLAB implementation of linear tetrahedral FEM with the Galerkin approach (Silvester and Ferrari, 1994; Kwon and Bang, 2000; Lionheart et al., 2004; Fernández-Corazza et al., 2013).

### 4.2. Simulation results

#### 4.2.1. non–ROI energy constraint

First, we solved the constrained directional maximization problem in Eq. (3) using the iterative SDPT3 solver (Toh et al., 1999) included in the CVX package (Grant and Boyd, 2014) for a wide range of α_I_ values, considering total current limit constraint in Eq. (3.ii) as $i_{max}=1\text{mA},$ and current limit per electrode constraint in Eq. (3.iii) as ${\tilde{i}}_{\max}=i_{max}1\;\text{and}\;{\tilde{i}}_{\min}=-i_{max}1.$

For each optimal solution $\hat{i}$ of the spanned α_I_ range, we computed (a) the integral of the electric field over *Ω*_ROI_ (i.e., the maximized functional **d^T^ΓTi** of Eq. (3)): normalized by the *Ω*_ROI_ volume, (b) the used budget, i.e., $\|\tilde{i}{\|}_{1},$ and (c) a focality metric. We defined focality as the ratio between the mean intensity at *Ω*_ROI_ to the square root of the mean energy at *Ω*_non–ROI_:

(14) $$\text{Integral focality}=\frac{\frac{\left(d^{\text{T}}\Gamma T\hat{I}\right)}{\Omega_{\text{ROI}}\;\text{volume}}}{\sqrt{\frac{\left({\hat{I}}^{\text{T}}T^{\text{T}}\Gamma_{\text{non}-\text{ROI}}T\hat{I}\right)}{\Omega_{\text{non–ROI}}\;\text{volume}}}}$$

**Fig. 1.**
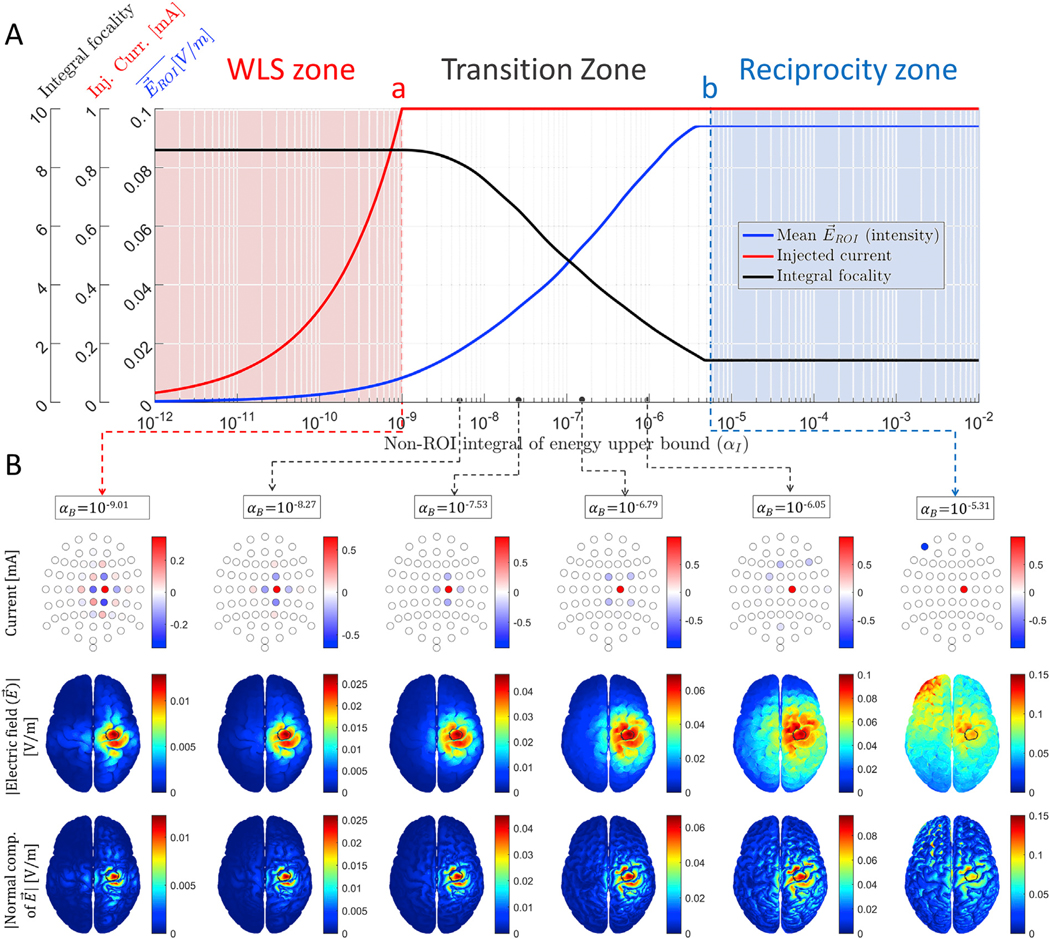
Iterative solutions to the constrained directional maximization problem in Eq. (3) with constraint of Eq. (3.i.a) and computed with the SDPT3 solver. (A) Mean ROI directional intensity measured as the functional to be maximized in Eq. (3) divided by *Ω*_ROI_ volume (blue line), total injected current (red line), and integral focality (black line) as a function of the *Ω*_non–ROI_ energy upper bound (α_I_). (B) Some examples of the iterative solutions: optimal current injection patterns $\hat{i}$ (first row); modulus of the electric field at the brain with *Ω*_ROI_ circled in black (second row), and absolute value of the normal-to-cortex component of the electric field (third row). Color scale limits are different, increasing from left to right. The solutions in the pale pink zone of (A) are equivalent, except for a scaling constant, to the WLS closed-form solution. The iterative solutions in the pale blue zone of (B) are equivalent to the closed-form one-to-one reciprocity solution. Between critical points “a” and “b”, there is a smooth transition between both extreme solutions.

There are several ways of defining focality, but we can group them in basically two types: as ratios between some ROI intensity and some non–ROI intensity (Cancelli et al., 2016; Wagner et al., 2016a), or as the radius of a sphere centered at the ROI containing some amount of total intensity (Dmochowski et al., 2011; Fernández-Corazza et al., 2016). We found the definition of Eq. (14) as the more natural definition according to the general problem in Eq. (3): the ratio of the expression to maximize in Eq. (3) to the constraint in Eq. (3.i.a). As the constraint is quadratic and the functional to maximize is linear with respect to $\hat{i},$ we applied the square root to the denominator (this also makes the metric to be unitless). Note that the integral focality in Eq. (14) can be interpreted as a ratio of the “therapeutic dose” to the “side-effects” where the larger is the better. In Fig. 1A, we plot the intensity, the amount of budget used and the integral focality as a function of α_I_. Fig. 1B depicts some examples of optimal current injection patterns and their resulting electric fields in the brain for evenly spaced and representative values of α_I_, and Movie M1 shows them for all the evaluated values of α_I_.

Supplementary video related to Fig. 1 can be found at https://doi.org/10.1016/j.neuroimage.2019.116403.

In Fig. 1A, three zones can be distinguished by different background colors. In the left zone (pale pink), the used budget is less than 100% of the allowed budget, *Ω*_non–ROI_ energy constraint in Eq. (3.i.a) dominates, and the total current limit constraint in Eq. (3.ii) has no influence on the solution. In the right zone (pale blue), the optimal solution remains the same regardless the value of α_I_, both the used budget and the maximum electric field at *Ω*_ROI_ are saturated, the total current limit constraint in Eq. (3.ii) dominates, and the *Ω*_non–ROI_ energy constraint in Eq. (3.i.a) has no influence. Lastly, in the middle zone (white background), the current budget is saturated, but a more intense electric field is delivered to *Ω*_ROI_ at the expense of a larger electric field energy at *Ω*_non–ROI_ (by increasing α_I_). The focality-intensity trade-off is clearly observed between critical points “a” and “b” (see Movie M1).

We then computed the closed-form WLS and reciprocity solutions following Eqs. (5) and (13), respectively. Supplementary Fig. S1 depicts injection patterns and their resulting electric fields obtained with the optimally scaled WLS formulation (left column) and with the one-to-one reciprocity optimization approach (right column). It is observed that they are indeed identical to the first and last columns of Fig. 1B, respectively.

An interesting observation from Fig. 1 is that, for a specific value of α_I_, the optimal solution obtained iteratively is equivalent to the WLS solution with the *ℓ*_1_ constraint (row 4 of Table 1 and Eq. (7a)). This finding verifies the equivalence of Eqs. (7a) and (7b) in Section (3.2.1). As we can determine the exact point that makes these two problems identical, we show both identical solutions in the first two columns of Supplementary Fig. S2. In the last two columns of Fig. S2, we show an example of verification of the equivalence between Eqs. (8a) and (8b), where the current limit per electrode constraints of Eq. (3.iii) are also considered.

We also obtained the iterative solutions of Eq. (3) in the right pale blue zone (large *α*), but setting different current limits per electrode (constraint in Eq. (3.iii)): first, setting ${\tilde{i}}_{\max}=i_{max}1\;\text{and}\;{\tilde{i}}_{\min}=-i_{max}/(L-1)1,$ and second, setting ${\tilde{i}}_{\max}=\left(i_{max}/2\right)1\;\text{and}\;{\tilde{i}}_{\min}=\;\left(-i_{max}/20\right)1.$ In Supplementary Fig. S3 we show that the optimal iterative solutions for these two cases are equivalent to the “1 source - (*L* − 1) sinks” and “2 sources - 20 sinks” closed-form reciprocity-based solutions introduced phenomenologically in Fernández-Corazza et al. (2016), thus verifying what we derive in Section 3.3.1.

#### 4.2.2. non–ROI elementwise intensity constraint

We also solved Eq. (3) but limited the electric field intensity at each *Ω*_non–ROI_ element (Eq. (3.i.b)) instead of limiting the integral of electric field energy (Eq. (3.i.a)) to compare with the previous approach. We set the constraint α_E_ to be equal for all mesh elements, which can be interpreted as limiting the maximum intensity in *Ω*_non–ROI_. We defined the most natural focality metric for this case as the ratio between the mean intensity at *Ω*_ROI_ to the maximum intensity at *Ω*_non–ROI_:

(15) $$\text{Elementwise focality}=\frac{\text{mean directional intensity over}\;\Omega_{\text{ROI}}}{\text{maximum intensity in}\;\Omega_{\text{non}-\text{ROI}}}\;\;=\frac{\frac{\left({\text{d}}^{\text{T}}\Gamma \hat{T}\hat{i}\right)}{\Omega_{\text{ROI}}\;\text{volume}}}{\underset{n\in \Omega_{\text{non}-\text{ROI}}}{\max}\left\|T_{n}{\hat{i}}_{2}\right\|}$$

**Fig. 2.**
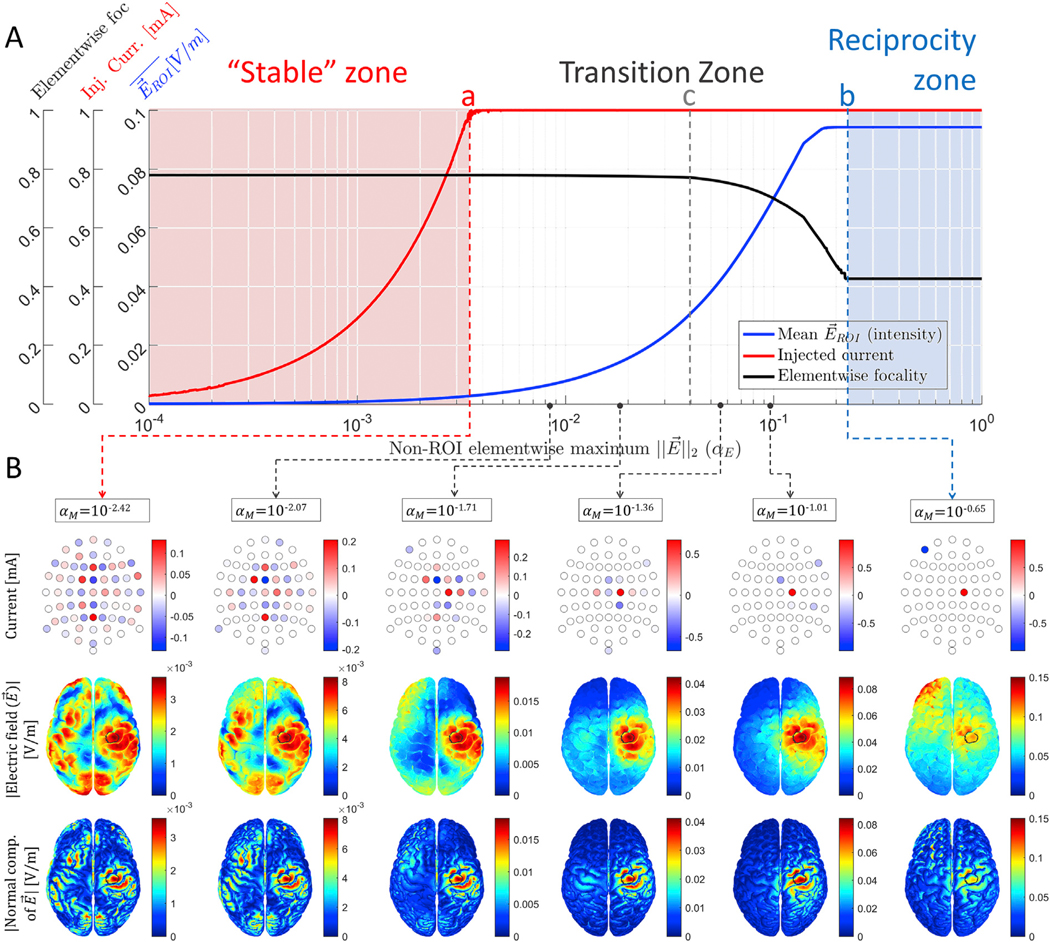
Iterative solutions to the constrained directional maximization problem in Eq. (3) with the constraint of limiting the electric field intensity at each *Ω*_non–ROI_ element (Eq. (3.i.b)). (A) Mean ROI directional intensity measured as the functional to be maximized in Eq. (3) divided by *Ω*_ROI_ volume (blue line), total injected current (red line), and elementwise focality (black line) as a function of the *Ω*_non–ROI_ maximum electric field (α_E_). (B) Some examples of the optimal solutions: optimal current injection patterns $\hat{i}$ (first row); modulus of electric field at the brain with *Ω*_ROI_ circled in black (second row), and absolute value of the normal component of the electric field (third row). Color scale limits are different, increasing from left to right. The solutions in the pale pink zone of (A) have the same pattern, except for a scaling constant. The iterative solutions in the pale blue zone of (B) are equivalent to the closed-form one-to-one reciprocity solution. Between critical points “a” and “b”, there is a smooth transition between both extreme solutions. We marked an additional point “c”, where the focality starts to decrease more sharply.

Fig. 2A shows the intensity, injected current and elementwise focality plots as a function of α_E_: Fig. 2B depicts some examples of the resulting optimal patterns and the imprinted brain electric field, and Movie M2 shows the full evolution of the optimal solutions.

Supplementary video related to Fig. 2 can be found at https://doi.org/10.1016/j.neuroimage.2019.116403.

When comparing Figs. 1 and 2, and Movies M1 and M2, the general behavior of a “scalable” solution on the leftmost zone, an intermediate transition zone, and a right zone resembling the one-to-one reciprocity solution holds for both optimization approaches. Note that the variety of solutions in the transition zone is not as rich as in the previous case. Also note that the extreme optimal solution for α_E_ < *a* looks somehow unintuitive as a large portion of *Ω*_non–ROI_ has intensities of the same order of magnitude as in *Ω*_ROI_. We present related discussion about this unintuitive pattern in Section 5.1.3 and Fig. S4. In that figure, we compared this unintuitive pattern with the WLS solutions and show that they are worse than this pattern in terms of the elementwise focality metric.

#### 4.2.3. Focality comparison

**Fig. 3.**
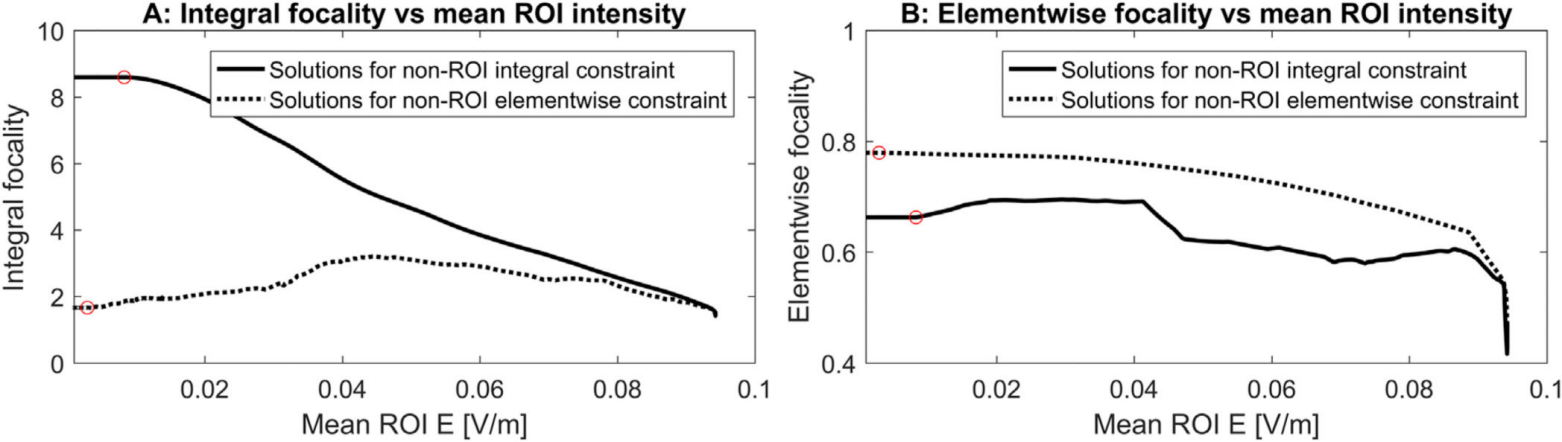
Focality values as a function of the mean electric field intensity in *Ω*_ROI_ for the solutions obtained with the *Ω*_non–ROI_ integral constraint (solid line) and with the *Ω*_non–ROI_ elementwise constraint (dotted line). Subfigure A shows the integral focality plots and subfigure B shows the elementwise focality plots for both optimal solution approaches. The red circles indicate the corresponding critical points “a” of Figs. 1A and 2A, i.e. the points where the optimal solutions reach the maximum available budget.

Figs. 1A and 2A are not directly comparable because the x-axis has different values in each case. To compare both cases it is necessary to plot the cross-focality metrics, i.e., the integral focality for the solutions obtained when imposing the *Ω*_non–ROI_ elementwise electric field constraint, and the elementwise focality for the solutions obtained when imposing the *Ω*_non–ROI_ integral constraint. In Fig. 3A we depict the integral focality values of the solutions obtained with both constraints and in Fig. 3B we plot the elementwise focality values for the same solutions. To make the comparison clearer, the focality values are plotted as a function of the mean electric field in *Ω*_ROI_.

In Fig. 3A, for ROI mean intensities larger than ~0.075 V/m, the integral focality obtained with the elementwise electric field restriction is almost as good as when optimizing for the energy integral. For ROI mean intensity values between ~0.04 V/m and ~0.075 V/m, the focality obtained with the elementwise constraint grows for lower ROI intensity values but less than the “natural” focality. Below ~0.04 V/m the focality for the solutions obtained with the elementwise constraint decrease with decreasing ROI intensity. Note that ~0.04 V/m corresponds to the inflection point in the focality plot marked as “c” in Fig. 2. Fig. 3B shows that the elementwise focality obtained with the integral energy constraint is about 30% lower than when using the elementwise constraint in almost all the evaluated range. Interestingly, for lower values of ROI mean intensity, the cross-focality (solid line) gets better, contrary to what happens in the dotted line of Fig. 3A for values lower than ~0.04 V/m.

### 4.3. Data and code availability statement

No prospective data were used for the research described in this article. The code supporting the findings of this study is available from the corresponding author upon request.

## 5. Discussion

### 5.1. Links between existing optimization algorithms

We theoretically proved that the apparently unrelated LS, WLS, and reciprocity-based solutions all belong to the same family of the general constrained maximizing intensity problem solutions of Eq. (3), constituting a unified approach (Sections 3.2 and 3.3). We also proved that constrained LS and WLS (Section 3.2.1) as well as constrained reciprocity (Section 3.3.1) are covered by the unified formulation. Even expanding “ring” configurations are also part of the same family, although this last finding was empirical. All these links were not fully noticed before, and it is the major novelty of this work.

An interesting finding is the existence of critical points “a” and “b” in all studied cases. For non–ROI electric field bounds (α_I_ or α_E_) lower than “a”, all iterative solutions are identically shaped, no matter if the restriction is for the energy integral over *Ω*_non–ROI_ (Eq. (3.i.a)) or elementwise (Eq. (3.i.b)). This is the pale pink zone in Figs. 1 and 2, where constraint in Eq. (3.i) is active and constraint in Eq. (3.ii) is inactive. In the case of restricting the integral, the optimal injection pattern shape is equivalent to the LS or WLS solution, but without exploiting the full current injection budget. Thus, an important result of this work is that the LS or WLS closed-form solutions, artificially scaled such that the total current budget is exploited (“scaled LS” in Table 1), is a simple way of obtaining the solution at point “a”. In the case of restricting the electric field elementwise (Eq. (3.i.b)), all iterative solutions for α_E_ values lower than “a” also have the same shape and different scale (see Movie M2) but we found this effect empirically and did not link them to a closed formula. In all cases, the solution at critical point “a” is the optimal solution in the sense that it exploits the full available budget and has the best focality,^7^ although, at the same time, it has the lowest ROI intensity and low sparsity requiring more active electrodes.

For α_I_ or α_E_ values larger than “a” and lower than “b”, the total current limit shapes the solutions producing a smooth transition towards reciprocity solutions when increasing (relaxing) the non–ROI intensity limit. This is the white background zone in Figs. 1 and 2, where both constraints of Eq. (3.i) and (3.ii) are active. A rich variety of optimal solutions consisting of expanding radius ring-shaped patterns occur naturally in this transition zone, which are more clearly seen in the first studied case (Fig. 1 and Movie M1). Many solutions in this middle zone resemble the *ad-hoc* “ring” patterns of previous studies (Datta et al., 2009; Dmochowski et al., 2011; Kuo et al., 2013; Fernández-Corazza et al., 2016). Thus, we computationally found that these “ring” solutions are also optimal. The well-known focality-intensity trade-off can be clearly observed and quantified in this transition zone, which is the zone with most practical interest. Moreover, we proved and verified that this middle zone also contains the WLS with limited total current injection optimal solution, either considering or not the current limit per electrode constraints (Supplementary Fig. S2).

Once critical point “b” is reached, the optimal solutions collapse into one of the reciprocity-based optimal solutions and remain identical for larger α_I_ or α_E_ values. This is the pale blue zone on the right part of Figs. 1 and 2, where constraint in Eq. (3.i) is inactive and constraint in Eq. (3.ii) is active. When not considering a current limit per electrode (Eq. (3.iii)), solutions in this zone are equivalent to the one-to-one reciprocity solution. In this case, the source and sink electrodes are selected directly as the nearest electrodes to the most positive and most negative forward projection EEG “poles” respectively. Moreover, we proved in section 3.3.1 that by setting different current limits per electrode in constraint of Eq. (3.iii), iterative solutions to Eq. (3) agreed exactly with the other rather intuitive variations of the “opposite” reciprocity solutions described in our previous work (Fernández-Corazza et al., 2016), proposed there with the aim of spreading out the typically undesired electric field concentration due to a low number of sinks. Thus, these solutions are not *ad-hoc*, but part of the set of solutions of the optimization problem in Eq. (3) (see Supplementary Fig. S3).In such cases, multiple current sources and sinks are selected as the electrodes with maximum differences in the virtual EEG forward projection potential. Overall, the solutions at critical points “b” exploit full available budget, have the highest ROI intensity and sparsity (requiring the minimum number of active electrodes), but the lowest focality.

#### 5.1.1. Focality

Many definitions of focality have been proposed in the literature so far. In this work, as we used different optimization criteria, we defined focality in the most natural way for each specific approach according to our understanding: the functional to maximize divided by the *Ω*_non–ROI_ electric field constraint. We also computed the “unnatural” or “cross” focality metrics, shown in Fig. 3. In this figure, it can be verified what is expected, that each focality definition is better for the solutions of its corresponding problem for any given ROI electric field intensity. Each optimal solution performance in terms of its cross-focality definitions might be of interest for a specific stimulation scenario and pre-treatment planning. For instance, by inspecting Fig. 3, it is possible to quantify how much of a focality metric is deteriorated when using a different optimization method than the “natural” one for this particular focality definition.

If a different focality metric is proposed to better quantify the trade-off between the wanted versus the unwanted effects, the problem statement should be changed accordingly to maximize whatever is defined as “wanted effects” constraining it to whatever is defined as “unwanted effects”. For example, we found that although directional electric field is maximized, the modulus of the electric field at *Ω*_non–ROI_ is typically limited. Moreover, the modulus of the electric field or the current density is typically depicted in most publications about TES optimization. We find this practice somehow contradictory. Therefore, besides depicting the modulus, we also depict the normal-to-cortex component in Figs. 1, 2, S1, S2, and S3. The directional optimization methods studied in this work result in much better focality when only considering normal-to-cortex component instead of the modulus. Note that some of the undesired *Ω*_non–ROI_ “hot-spots” seen when depicting the modulus are being targeted tangentially to the orientation assumed as physiologically influential. Thus, a new possible optimization method with its corresponding natural focality definition might be maximizing the directional intensity (as we did in this work), but constraining just the projected component of the electric field to the orientation assumed as physiologically influential at the *Ω*_non–ROI_ elements instead of the electric field modulus. Typically, it is assumed that only the normal to the cortical surface electric fields affect the brain, but this is still an open question.

#### 5.1.2. Previous studies placed into the unified context

According to the patterns shown in Figs. 3 and 4 of (Guler et al., 2016a), iterative solutions resemble the sparse reciprocity “opposite” solutions involving 6–7 electrodes as sources and the same number as sinks. Effectively, they adopted a non–ROI energy bound of 10^−6^ A^2^/m, which corresponds to a loose constraint for their equivalent to our α_I_ value according to their Fig. 6. This value of the non–ROI energy bound brings a solution into the reciprocity zone. The constraint on current per electrode defined roughly the number of active electrodes. The top part of their Fig. 6 (large α_I_) is equivalent to the right “pale blue” reciprocity zones of Figs. 1A and 2A, and the bottom part (low α_I_.) is equivalent to the left “pink” WLS zone of Fig. 1A. In Fig. 7 of Guler et al. (2016a), a different (much lower) non–ROI energy bound value is used to get non-sparse LS-like solutions resembling the constrained LS solutions by Dmochowski et al. (2011) and Ruffini et al. (2014).

#### 5.1.3. WLS vs LS

In Section 3.2, we showed that the integrals in Eq. (1) can be formulated as a version of WLS where the weights are the volumes of each finite element. However, in most TES optimization approaches using FEM meshes, this weighting by element volume was not considered (e.g. Dmochowski et al., 2011; Ruffini et al., 2014; Cancelli et al., 2016; Fernandez-Corazza et al., 2016) - including our previous work - deriving in unweighted LS. In FEM, the element volumes might vary significantly from each other, thus we believe that the WLS version with the weighting matrix containing the element volumes is more appropriate than the unweighted LS. Of course, additional weighting matrices can also be considered in addition to the volume weighting matrix **Γ**.

#### 5.1.4. Elementwise non–ROI intensity constraint

In Fig. 2B, the lower extremal solutions for α_E_ < a do not look optimal in terms of focality. This is because, based on our intuition, we typically think of the focality metrics in terms of the integral focality definition. In Supplementary Fig. S4, we verify that indeed this rather unintuitive solution is optimal in terms of elementwise focality for this example, which is the most natural focality definition for constraint in Eq. (3.i.b). Note that if the elementwise maximum intensity is restricted, there is a very sharp transition imposed at the Ω_ROI_ boundary between a “free” and a “restricted” element. If α_E_ is too small, on the boundary it is not possible for the electric field to follow this large jump between a “free” ROI element and a non–ROI constrained element right next to it. If α_E_ increases, this restriction is loosened allowing a softer transition between ROI and non–ROI neighboring elements. Note that this sharp restriction is not necessarily imposed when restricting the non–ROI integral of the electric field. An interesting observation is that these rather “unintuitive” solutions between points marked as “a” and “c” produce no improvement in focality and thus, have little practical interest. And at point “c”, the optimal solution looks qualitatively more focal also in terms of integral focality than the solution at point “a” (see Fig. 2). The most interesting region is thus between points “c” and “b”, where the intensity-focality trade-off is more visible.

### 5.2. Practical applications of this work

The way we present the focality-intensity trade-off in Figs. 1 and 2 should help a TES-planner to better define what α value (either α_I_ or α_E_) to select for a specific ROI, head model, and focality criteria depending on the specific application requirements. This focality-intensity visualization approach is also a novelty of this work.

For instance, it is possible to quantify how much intensity or focality is lost for each value of α at intermediate values within the “a” - “b” range. If a minimum intensity threshold is defined for a desired ROI, a TES-planner can search for the lowest α value with this intensity, quantify how much focality is lost, select this α value to feed the optimization problem and use the optimal solution to stimulate that ROI. One can also compare critical points “a” and “b” scanning ROIs placed at different brain locations to generate spatial brain maps of maximum possible focalities or doses. Even more, one can study how much these focality and intensity bounds are influenced by model parameters such as the skull conductivity for example. It might also be interesting to analyze how the gap between critical points “a” and “b” varies for superficial versus deep ROIs.

Another practical use is the analysis of the one-to-one reciprocity approach that resembles the anodal-cathodal current injection pattern, probably the most frequently used pattern in TES. If placed at the EEG “poles”, the anodal-cathodal montage is the extreme solution of the maximal intensity and the lowest focality on the target. This pattern can produce unwanted large amounts of stimulation near the cathodal electrode. In order to reduce this unwanted effect, a potential TES user can evaluate the possibility of using the two possible alternatives we presented: selecting a lower α value or choosing a lower value of $\left|{\tilde{i}}_{\text{min}}\right|.$ The first alternative can be seen in Figs. 1 and 2, where lower values of α reduce large intensities near the sink. For the second alternative, we show, in the first two columns of Supplementary Fig. S3, an example of selecting ${\tilde{i}}_{\min}=-i_{max}/(L-1)1,$ where it is effectively observed how the unwanted stimulation near the sink is drastically reduced. The interesting aspect of this work is that these solutions can be derived and analyzed from the general framework of Eq. (3).

Our findings suggest that a sliding bar for selecting the non–ROI constraint (α_I_ *or* α_E_ ) can be included in neurostimulation planning software to span the whole range of optimal solutions. Moreover, within the same framework, a disconnected ROI can be selected to target multiple brain regions simultaneously as it can be useful, for instance, to alter large-scale brain networks. Also, different restrictions can be imposed for anatomically specific non–ROIs with different sensitivity (for instance, visual nerves) by partitioning constraints (3.i.a) or (3.i.b) into subdomains of *Ω*_non–ROI_.

Although the analysis done in this work is based in the TES context, the same results can be applied to other techniques, for instance, to multi-electrode intracranial electrical stimulation using electrocorticography (ECoG), or to deep brain stimulation with stereo-EEG electrodes (Guler et al., 2018). In these applications, the electric field intensities can be much larger than in TES due to electrode proximity to brain tissue and thus, the focality might be prioritized. Another technique that can benefit from this work is the application of high frequency alternating electric fields to treat malignant glioma, known as TTFields (Miranda et al., 2014; Wong et al., 2015). In this application, the goal is to inject currents that cover all possible orientations at the localized tumor. An evenly distributed set of orientations can be defined as different targets within the tumor, and the TES optimization problems can be solved for each target to determine a set of current injection patterns that improves the spatial and directional coverage. The reciprocity principle also holds for the magnetic field and a duality like this one can be found for TMS/MEG, although dual TMS/MEG equipment is technically much more complex to build.

#### 5.2.1. Experimental findings

There have been some studies that experimentally tested individually optimized TES montages and/or compared different solutions covered in this work. The reciprocity based TES targeting method has been used by Luu et al. (2016) in a pulsed TES study of the subject-specific motor area with a restricted number of electrodes, eight sources and eight sinks. They found significant effects compared to sham controls, though no comparison with a standard montage or other optimization methods was made. Dmochowski et al. (2013) and Richardson et al. (2015) did an experimental post-stroke rehabilitation study comparing the optimal directional maximization with the total current constraint using two sources and two sinks (that we showed here to be equivalent to the reciprocity method) and a conventional two-patch tDCS electrode placement. They found a better outcome with the optimized pattern, but with no statistical significance.

Cole et al. (2018) studied the effects of high definition (HD) tDCS (a ring shaped montage with one source and four sinks) and conventional tDCS on motor learning in children and they found differences with respect to sham controls but no significant differences between the two tested montages. Kuo et al. (2013) compared HD and conventional tDCS with an experiment stimulating the motor cortex and they found that neuronal plasticity changes showed a more delayed peak and longer lasting after-effects after HD tDCS, as compared to conventional tDCS. Jacquemin et al. (2018) reported no significant differences in the effects of electrical stimulation in tinnitus patients comparing conventional versus HD tDCS. In these studies, the placement of HD tDCS electrodes was based on standardized atlases or templates but not on subject specific optimal locations.

Fischer et al. (2017) compared a classical tDCS montage stimulation targeting a single brain area with a multifocal optimized stimulation based on resting state fMRI networks, finding that multifocal network targeting increased the M1 excitability when compared to traditional single ROI stimulation. More recently, Laakso et al. (2019) found a correlation between the modelled electric field intensity and the efficacy of tDCS in a motor evoked potential experiment. This finding suggests that the inter-subject variability might be explained by differences in individual electric fields and thus, individual optimization patterns should improve individual TES efficacy.

The major conclusion of these studies is that individual head modeling and TES optimization are very important to produce individual optimal patterns that can possibly account for the variability of experimental results. More comparative studies are still needed to assess the true efficacy of the full range of possible individualized optimal solutions, from critical point “a” to critical point “b” in Figs. 1 and 2.

### 5.3. Open debates

We believe that the question of whether focality or intensity (total or directional) should be prioritized for each application is still an open question. Each algorithm is optimal in a different sense, and the spirit of this work is to be impartial to all presented algorithms without judging them by their neurophysiological efficacy. Quantitative analysis of these algorithms in terms of focality, intensity and other performance metrics for clinical applications is out of the scope of this work, as this was done at length elsewhere (Datta et al., 2009; Dmochowski et al., 2011; Datta et al., 2013; Dmochowski et al., 2013; Ruffini et al., 2014; Fernández-Corazza et al., 2015; Cancelli et al., 2016; Fernández-Corazza et al., 2016; Wagner et al., 2016a).

A question of which orientation is better to target in TES, i.e. which one is more physiologically influential, is still in debate and would depend on the specific application. If pyramidal neurons are the target, a stimulation perpendicular to the cortex surface should be preferred, whereas if interneuron synapses are aimed, a tangential-to-cortex stimulation would be more appropriate. Note that all covered algorithms in this work are applicably for any orientation of choice. We opted to use normal-to-cortex orientation to illustrate our results because it is the most commonly used orientation.

## Supplementary Material

1

2

3

## Figures and Tables

**Table 1:** Summary of covered approaches in the unified framework.

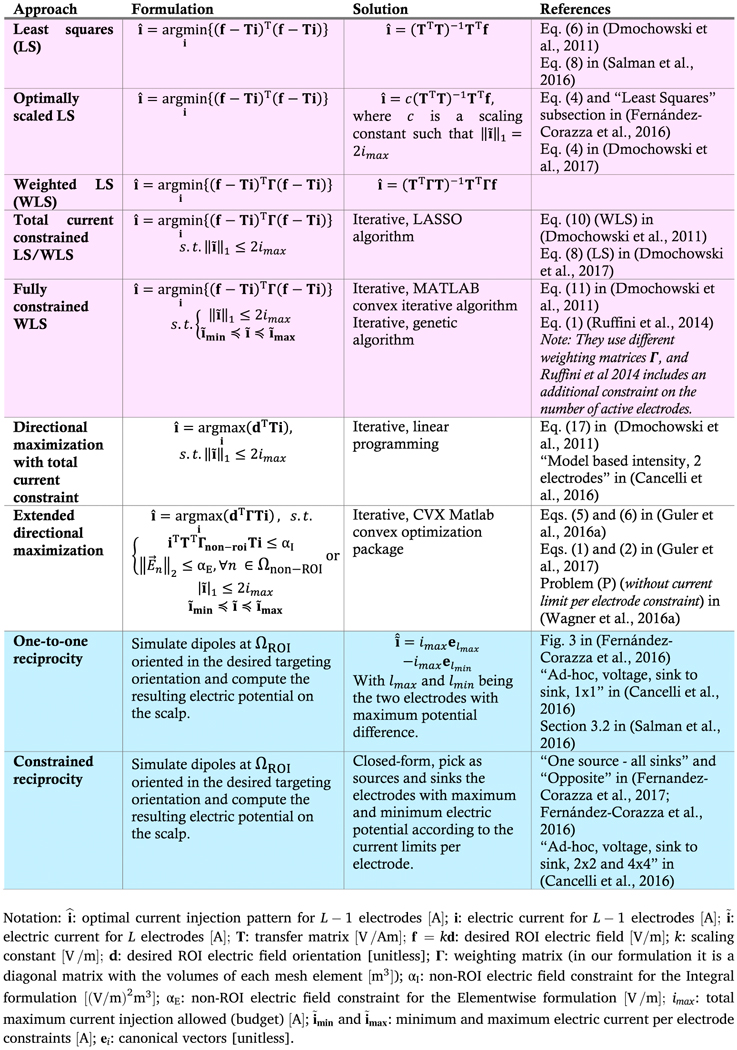			

**Fig. B1.**
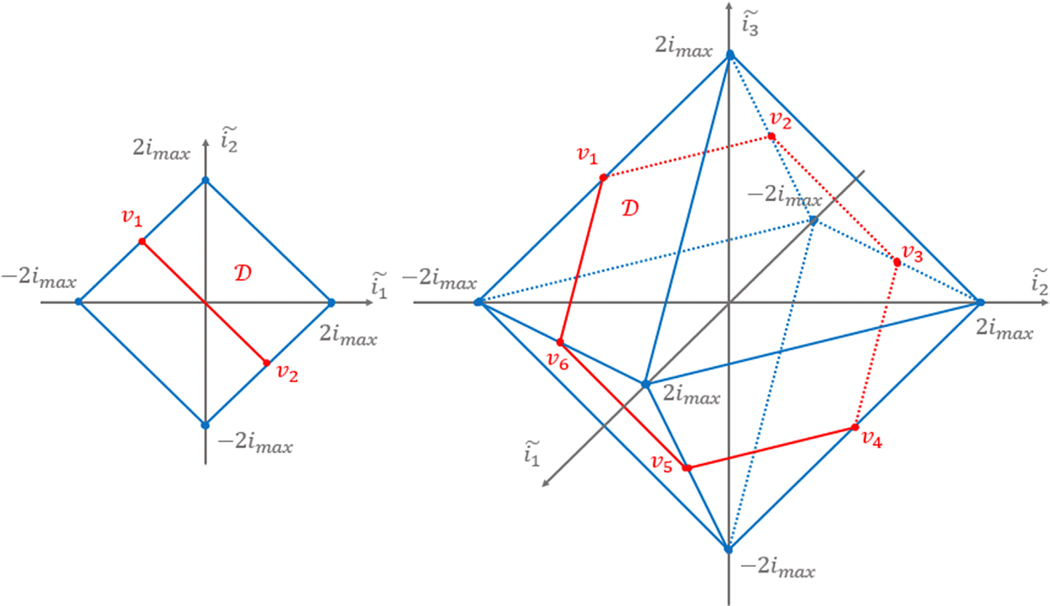
Feasible domains D for the 2D (left) and 3D (right) cases, assuming two and three electrodes respectively. All vertices correspond to only two active electrodes. The 2D case is trivial, with two electrodes, there are only two possible injection patterns: $\left(i_{max},-i_{max}\right)\;\text{and}\;\left(-i_{max},i_{max}\right).$ For the 3D case there are six vertices, i.e., six possible solutions.

**Fig. B2.**
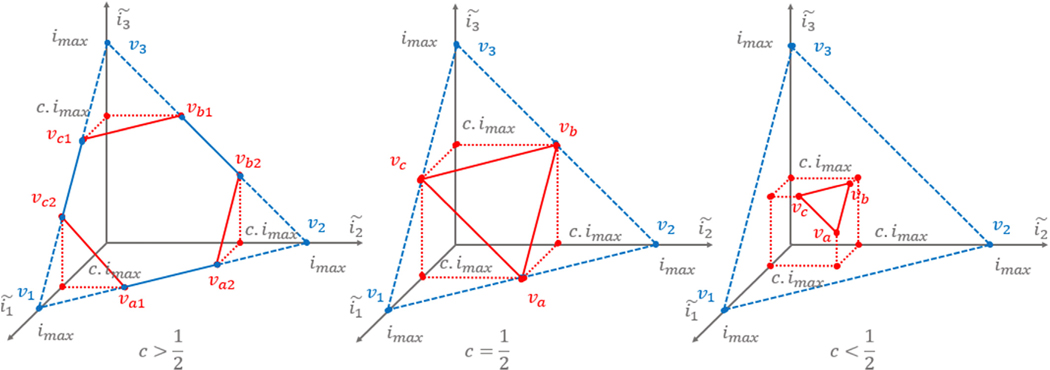
Example of how the standard simplexes in Eq (B.3) are altered with a current limit per electrode lower in absolute value than *i*_*max*_. In this example we set $c>\frac{1}{2}(\text{left}),c=\frac{1}{2}\;(\text{center}),\;c<\frac{1}{2}\;(\text{right}).$ Corners *v*_1_; *v*_2_; *v*_3_ do not longer belong to the truncated simplex, and points *v*_*a*_; *v*_*b*_; *v*_*c*_ are new corners and thus, possible solutions.3

## Footnotes

1 To be more precise, the functional to maximize in Eq. (3) should be $d_{\text{ROI}}^{\text{T}}\Gamma_{\text{ROI}}T_{\text{ROI}}i,$ where subscript “ROI” means “trimmed to ROI elements”, because the integral of Eq. (1) is taken over *Ω*_ROI_. But, as the elements of **d** corresponding to *Ω*_non–ROI_ are set to zero and **Γ** is a diagonal matrix, it is equivalent to use $d_{\mathrm{ROI}}^{\text{T}}\Gamma_{\mathrm{ROI}}T_{\mathrm{ROI}}i\;\text{or}\;d^{\text{T}}\Gamma Ti.$

2 The approximation error is proportional to the product of average intensity at *Ω*_ROI_ times the *Ω*_ROI_ volume which is typically much smaller than the product of average intensity at *Ω*_non–ROI_ times the *Ω*_non–ROI_ volume.

3 In mathematical optimization, the KKT conditions are the necessary conditions for a solution in nonlinear programming to be optimal. If the problem is convex, these conditions are also sufficient (Boyd and Vandenberghe, 2004).

4 In the LS/WLS approaches here and in Table 1, a target vector **f**(or *k***d**) might have non-zero values at *Ω*_non–ROI_. However, in LS/WLS it is also common practice to set the desired electric field to zero at *Ω*_non–ROI_. For simplicity, we also adopt this practice for **f**and **d** throughout the manuscript.

5 These vectors correspond to the canonical basis.

6 To exactly reproduce solutions from (Fernández-Corazza et al., 2016), **Γ** in Eq. (10) should be set as the identity matrix as in our previous work we didn’t consider integration in the objective function.

7 For each case natural definition of focality.

## Appendix A

### Appendix A.1. Equivalence between Eqs. (4) and (6)

Proof that the QCLP in Eq. (4) has the solution of Eq. (6). The standard form of this QCLP is $\hat{i}=\underset{i}{\mathrm{argmin}}\left(-d^{\text{T}}\Gamma Ti\right)\;s.t.\;i^{\text{T}}T^{\text{T}}\Gamma Ti-\alpha \leq 0.\;\text{Optimal}\;\hat{i}\;\text{and}\;\hat{\text{λ}}$ must satisfy the Karush-Kuhn-Tucker (KKT) conditions (Boyd and Vandenberghe, 2004):

(A.1.a) $$i^{\text{T}}T^{\text{T}}\Gamma Ti-\text{α}\leq 0$$

(A.1.b) λ≥0

(A.1.c) $$\lambda \left(i^{\text{T}}T^{\text{T}}\Gamma Ti-\alpha \right)=0$$

(A.1.d) $$\frac{\partial \left(-d^{\text{T}}\Gamma Ti\right)}{\partial i}+\text{λ}\frac{\partial \left(i^{\text{T}}T^{\text{T}}\Gamma Ti-\text{α}\right)}{\partial i}=-d^{\text{T}}\Gamma T+\text{λ}2i^{\text{T}}T^{\text{T}}\Gamma T=\bar{0}$$

Clearing iT from Eq. (A.1.d) we get

(A.2) $$i^{\text{T}}=\frac{d^{\text{T}}\Gamma T{\left(T^{\text{T}}\Gamma T\right)}^{-1}}{2\text{λ}}$$

From Eq. (A.1.c) either *λ* = 0 or **i**^T^**T**^T^**ΓTi** = α. When *λ* = 0, since **Γ** is not 0, from the fourth condition the desired orientation field **d** should be null, which is uninteresting, so *λ* ≠ 0 and **i**^T^**T**^T^**ΓTi** = α. This means that optimal solution lies on the edge of the feasible set, as expected in a QCLP. Replacing **i** from Eq. (A.2) into **i**^T^**T**^T^**ΓTi** = α, we get:

(A.3) $$\lambda =\frac{1}{2}\sqrt{\frac{d^{\text{T}}\Gamma T{\left(T^{\text{T}}\Gamma T\right)}^{-1}T\Gamma d}{\text{α}}}.$$

Replacing *λ* in Eq. (A.2) with Eq. (A.3), we obtain the solution of Eq. (6). The same procedure holds if **Γ**_**nr**_^8^ is used instead of **Γ** as in the constraint of Eq. (4). Note that *λ* is in [m/V].

### Appendix A.2. Equivalence between Eqs. (7a) and (7b)

We now prove that Eqs. (7a) and (7b) have the same KKT conditions for $\alpha^{\prime }={\hat{i}}^{\text{T}}T^{\text{T}}\Gamma T\hat{i},$ and thus, the problems are equivalent. First, we replace the ℓ_1_-norm constraint $\|\tilde{i}{\|}_{1}\leq 2i_{max}$ by an equivalent linear set of constraints. The points in the L-dimensional space determined by this constraint are the interior points of an L-dimensional orthoplex or hyperoctahedron (the extension of a square with vertices $\left\{\left(\pm 2i_{max},0\right),\left(0,\pm 2i_{max}\right)\right\}$ in 2D and of a octahedron with vertices $\left\{\left(\pm 2i_{max},0,0\right),\left(0,\pm 2i_{max},0\right),\left(0,0,\pm 2i_{max}\right)\right\}$ in 3D). The faces of the orthoplex are all regular simplices (hyperplanes) scaled by 2*i*_*max*_. For instance, for the 3D case, we can rewrite $\|\tilde{i}{\|}_{1}\leq 2i_{max}$ as the set of constraints:

(A.4) $$\{\begin{array}{l} {\tilde{i}}_{1}+{\tilde{i}}_{2}+{\tilde{i}}_{3}\leq 2i_{max}\\ {\tilde{i}}_{1}+{\tilde{i}}_{2}-{\tilde{i}}_{3}\leq 2i_{max}\\ {\tilde{i}}_{1}-{\tilde{i}}_{2}+{\tilde{i}}_{3}\leq 2i_{max}\\ {\tilde{i}}_{1}-{\tilde{i}}_{2}+{\tilde{i}}_{3}\leq 2i_{max}\\ -{\tilde{i}}_{1}+{\tilde{i}}_{2}-{\tilde{i}}_{3}\leq 2i_{max}\\ -{\tilde{i}}_{1}+{\tilde{i}}_{2}-{\tilde{i}}_{3}\leq 2i_{max}\\ -{\tilde{i}}_{1}-{\tilde{i}}_{2}+{\tilde{i}}_{3}\leq 2i_{max}\\ -{\tilde{i}}_{1}-{\tilde{i}}_{2}-{\tilde{i}}_{3}\leq 2i_{max} \end{array}\Rightarrow \underset{J}{\underset{⟹}{\left[\begin{array}{ccc} 1 & 1 & 1\\ 1 & 1 & -1\\ 1 & -1 & -1\\ 1 & -1 & -1\\ -1 & 1 & 1\\ -1 & 1 & -1\\ -1 & -1 & 1\\ -1 & -1 & -1 \end{array}\right]}}\tilde{i}≼2i_{max}1\Rightarrow \;\tilde{j}\tilde{i}≼2i_{max}1\Rightarrow JHi≼2i_{max}1,$$

where **J** is a new matrix that accounts for the eight faces of the octahedron, **H** is the matrix that does the conversion $\tilde{i}=H\cdot i,1$ is a vector with all ones, and ≼ means ≤ but elementwise. Note that first and last rows of **J** impose trivial constraints as the additional constraint $1^{\text{T}}\tilde{i}=0$ must hold, i.e. the sum of all elements of $\tilde{i}$ is zero by Kirchhoff’s current law.

Matrix **J** can be similarly built for the L-dimensional space as a large but linear set of 2^*L*^ constraints (or 2^*L*^ − 2 if trivial top and bottom constraints are removed), i.e. $\|\tilde{i}{\|}_{1}\leq 2i_{max}$ is equivalent to $J\tilde{i}≼2i_{max}1.$ The linear set of constraints as an alternative formulation for the *ℓ*_1_-norm constraint is useful to calculate the derivative in the last KKT condition. Indeed, the KKT conditions for Eq. (7.a) become:

(A.5.a) $$i^{\text{T}}T^{\text{T}}\Gamma Ti-\text{α}\leq 0$$

(A.5.b) $$JHi-2i_{max}1≼\bar{0}$$

(A.5.c) $$1^{\text{T}}Hi=0$$

(A.5.d) $$\lambda_{q}^{a}\geq 0$$

(A.5.e) $$\lambda_{l}^{a}≽\bar{0}$$

(A.5.f) $$\lambda_{q}^{a}\left(i^{\text{T}}T^{\text{T}}\Gamma Ti-\alpha \right)=0$$

(A.5.g) $$\lambda_{1}^{\text{aT}}\left(JHi-2i_{max}1\right)=\bar{0}$$

(A.5.h) $$-d^{\text{T}}\Gamma T+\lambda_{q}^{a}2i^{\text{T}}T^{\text{T}}\Gamma T+\lambda_{l}^{aT}JH+v^{a}1^{\text{T}}H=\bar{0}$$

Where $\lambda_{q}^{a}$ is the multiplier associated to the quadratic constraint of the first row, $\lambda_{l}^{a}$ is a vector with the 2^*L*^ multipliers associated to the linear inequality constraints of the second row, *v* is the multiplier associated to the linear equality constraint of the third row, and superscript “a” is to distinguish them to the multipliers of Eq. (7.b).

Similarly, the KKT conditions for Eq. (7.b) are:

(A.6.a) $$JHi-2i_{max}1≼\bar{0}$$

(A.6.b) $$1^{\text{T}}Hi=0$$

(A.6.c) $$\lambda_{l}^{b}≽\bar{0}$$

(A.6.d) $$\lambda_{l}^{b^{T}}\left(JHi-2i_{max}1\right)=\bar{0}$$

(A.6.e) $$-2kd^{\text{T}}\Gamma T+2i^{\text{T}}T^{\text{T}}\Gamma T+\lambda_{l}^{b^{T}}JH+v^{b}1^{\text{T}}H=\bar{0}$$

Where now the superscript “*b*” refers to Eq. (7.b). Note the slight difference of a “2” multiplying the first term of Eq. (A.6.e) and the lack of a multiplier in the second term of same equation. These two terms arise from taking the derivative with respect to i of the functional to minimize in Eq. (7b).^9^

Dividing Eq. (A.6.e) by 2*k*, both KKT set of conditions (A.5) and (A.6) are equivalent when $2k\lambda_{q}^{a}=1,2k\lambda_{l}^{a}=\lambda_{l}^{b},\;\text{and}\;2kv^{a}=v^{b}.\;\text{KKT}$ condition in Eq. (A.5.g) and $2k\lambda_{q}^{a}=1$ mean that $\lambda_{q}^{a}\neq 0$ and imply that $\alpha^{’}={\hat{i}}^{\text{T}}T^{\text{T}}\Gamma T\hat{i},\;\text{where}\;\hat{i}$ is the optimal solution to both problems. In our example, we solved Eq. (7.b) assuming *k* = 1V/m, used that solution to compute $\alpha^{’}={\hat{i}}^{\text{T}}T^{\text{T}}\Gamma T\hat{i}$ and verified that with this specific value, solution to Eq. (7.a) indeed is identical to solution to Eq. (7.b). We show this comparison in the first two columns of Supplementary Fig. S3.

### Appendix A.3. Equivalence between Eqs. (8a) and (8b)

Similarly to previous subsection, one can prove the equivalence of the KKT conditions for Eqs. (8a) and (8b) when $\alpha^{”\prime }={\hat{i}}^{\text{T}}T^{\text{T}}\Gamma T\hat{i},$ now with $\hat{i}$ being the optimal solution of the WLS problem in Eq. (8b). The difference with Eq. (7) is the addition of the set of constraints ${\tilde{i}}_{\min}≼\tilde{i}≼{\tilde{i}}_{\max}.$ These are simple linear constraints that can be easily incorporated to the KKT conditions of Eqs. (A.5) and (A.6). We validated this equivalence in an example and depict it in the last two columns of Fig. S3.

## Appendix B

### Appendix B.1. Proof that solutions to Eq. (12) have only 2 active electrodes

Here we prove that the corners of the feasible domain of Eq. (12) only have two active electrodes. We start by studying the intersection of the *ℓ*_1_-norm constraint for $\hat{i}$ and the Kirchhoff’s Law condition in Eq. (2). Thus, we can rewrite Eq. (12) as $\hat{i}=\underset{i}{\mathrm{argmax}}\left(\Phi^{\text{T}}i\right)\;s.t.\;\{\|\tilde{i}{\|}_{1}\leq 2i_{max}1^{\text{T}}\cdot \tilde{i}=0\;\text{where}\;\tilde{i}=Hi$ is the expanded current injection pattern considering all electrodes as defined in Eq. (2), and 1 is a vector with all ones and same length of $\hat{i}$. The *ℓ*_1_-norm constraint $\|\tilde{i}{\|}_{1}\leq 2i_{max}$ can be represented as an L-dimensional orthoplex or hyperoctahedron (see Appendix A). Fig. B1 shows the *ℓ*_1_-norm constrained domain in blue (square in 2D and octahedron in 3D) and the Kirchhoff’s Law geometrical representation for the 2D and 3D cases (two and three electrodes respectively) of expanded $\hat{i}$ in red.

The fact that the corners of the intersection of the two constraints are vertices where only two electrodes are active can be generalized to L dimensions as follows. The two constraints can be expressed as:

(B.1) $$\{\begin{array}{cc} \left(a\right) & \pm {\tilde{i}}_{1}\pm {\tilde{i}}_{2}\pm {\tilde{i}}_{3}\pm \ldots \pm {\tilde{i}}_{L-1}\pm {\tilde{i}}_{L}=2i_{max}\\ \left(b\right) & {\tilde{i}}_{1}+{\tilde{i}}_{2}+{\tilde{i}}_{3}+\ldots +{\tilde{i}}_{L-1}+{\tilde{i}}_{L}=0 \end{array}$$

where the ± sign changes for each orthant (n-dimensional generalization of 3D octant). First, note that the two hyperplanes in Eq. (B.1.a) bounding the two orthants with all positive and all negative signs are parallel to the hyperplane in Eq. (B.2.b). Thus, there is no intersection among them. Second, without loss of generality we analyze the hyperplane at the orthant where first *R* < *L* variables are positive and the rest are negative, i.e. condition in Eq. (B.1.a) is $+\;{\tilde{i}}_{1}+{\tilde{i}}_{2}+{\tilde{i}}_{3}+\ldots +{\tilde{i}}_{R}-{\tilde{i}}_{R+1}-{\tilde{i}}_{R+2}-\ldots -{\tilde{i}}_{L}=2i_{max}.$ By adding and subtracting this expression to Eq. (B.1.b) we obtain:

(B.2) $$\{\begin{array}{ll} \left(a\right) & \;{\tilde{i}}_{1}+{\tilde{i}}_{2}+{\tilde{i}}_{3}+\ldots +{\tilde{i}}_{R}=i_{max}\\ \left(b\right) & -{\tilde{i}}_{R+1}-{\tilde{i}}_{R+2}-\ldots -{\tilde{i}}_{L}=i_{max} \end{array}$$

The expressions in Eq. (B.2) are the positive and negative standard simplices (scaled to *i*_*max*_) of *R* and *L* − *R* dimensions respectively. It is known that the *D* + 1 vertices of the standard D-simplex are the points $e_{i}\in {\mathbb{R}}^{D+1}$ corresponding to the canonical basis vectors. Thus, the vertices of the intersection in Eq. (B.2) have the form: $i_{max}e_{i}-i_{max}e_{j},\;\text{for}\;i\in \{1,\ldots ,R\}\;\text{and}\;j\in \{R+1,\ldots ,L\}$ meaning that only two electrodes are active at each possible solution, finalizing the proof.

### Appendix B.2. Considering maximum current per electrode limit

We now consider additional constraints ${\tilde{i}}_{\min}≼\tilde{i}≼{\tilde{i}}_{\max},$ where the absolute value of every entry of ${\tilde{i}}_{\min}\;\text{and}\;{\tilde{i}}_{\max}$ is smaller than *i*_*max*_. For simplicity, let us assume first that we impose the same values *c.i*_*max*_ for all ${\tilde{i}}_{\max}$ elements and $-c.i_{max}$ for all ${\tilde{i}}_{\min}$ elements, with *c* being an arbitrary real scalar in the [0, 1] range. For visualization purposes, let us also assume that *R* = 3 in Eq. (B.2.a). Depending on the value of constant *c*, the simplex will be now truncated by the planes ${\tilde{i}}_{i}=c.i_{max},$ and six or three vertices will replace previous 3 vertices v1, v2 and v3, as depicted in Fig. B2 for different values of constant *c*.

If $c>\frac{1}{2}\;\text{or}\;c=\frac{1}{2},$ all corners are defined by two active (positive) electrodes and one inactive electrode with zero current. If $c<\frac{1}{2},$ three electrodes are active, two of them supplying the maximum possible current per electrode and the third one supplying the rest to inject a total of *i*_*max*_.

When *c* is small enough (in this example, if $c<\frac{1}{3}$), there is no possible solution on the simplex because the sum of the three positive injections can never be equal to *i*_*max*_. In this case, more electrodes (i.e. *R* > 3) are needed to inject *i*_*max*_. That is, a different orthant with more positive current injection electrodes (with *R* > 3) contains the optimal solution for the sources. As *c* gets smaller, more electrodes are involved in the possible solutions or corners, all of them injecting the total allowed current per electrode *c.i*_*max*_, except, possibly, for one that supplies the rest of the current to reach *i*_*max*_. The reasoning for imposing ${\tilde{i}}_{\min}$ limits to the sink electrodes is analogous. In the extreme case that $c<\frac{1}{L/2},$ half of the electrodes will be involved as sources and the other half as sinks, and the optimal solution will not touch any simplex determined by the $\|\tilde{i}{\|}_{1}\leq 2i_{max}$ constraint.

Among all corners or possible solutions, the optimal one is the one that maximizes $\Phi_{\Gamma }^{\text{T}}\;i$ (see Eq. (12)). It is then evident that the optimal solution involves the electrodes with maximum potential Φ_Γ_ differences. And all sources inject the same amount of current except, possibly, for one, and the same happens with the sinks.

Lastly, if the current injection bounds are not the same for all electrodes, the same reasoning can be applied and the optimal solution is found as follows: pick the electrode with largest electric potential and inject as much current as possible, then select the second electrode with maximum potential and inject as much current as possible, and repeat this process until the total maximum current injection limit is reached. Then, repeat this procedure for the sink electrodes but for the lowest Φ_Γ_. That one is the optimal solution to Eq. (12) with the addition of the ${\tilde{i}}_{\min}≼\tilde{i}≼{\tilde{i}}_{\max}$ set of constraints.

## Appendix C. Supplementary data

Supplementary data to this article can be found online at https://doi.org/10.1016/j.neuroimage.2019.116403.
