# Supplementary material for: Unification of optimal targeting methods in transcranial electrical stimulation
Source: Neuroimage. Author manuscript; Available in PMC 2020 Apr 1. (PMC7110419; doi:10.1016/j.neuroimage.2019.116403)
Supplement: 1 [file NIHMS1067608-supplement-1.docx]

Unification of optimal targeting methods in Transcranial Electrical Stimulation

Mariano Fernandez-Corazza^1*^, Sergei Turovets^2^ and Carlos Muravchik^1,3^.

^1^LEICI Instituto de Investigaciones en Electrónica, Control y Procesamiento de Señales, Universidad Nacional de La Plata - CONICET, Argentina

^2^NeuroInformatics Center, University of Oregon, Eugene, OR, USA

^3^Comisión de Investigaciones Científicas - CICPBA, Provincia de Buenos Aires, Argentina

* Correspondence: Mariano Fernández-Corazza, LEICI Instituto de Investigaciones en Electrónica, Control y Procesamiento de Señales, Facultad de Ingeniería, Universidad Nacional de La Plata, CC91 (1900), La Plata, Buenos Aires, Argentina.

E-mail: [marianof.corazza@ing.unlp.edu.ar](mailto:marianof.corazza@ing.unlp.edu.ar).

**Supplementary figures with captions.**


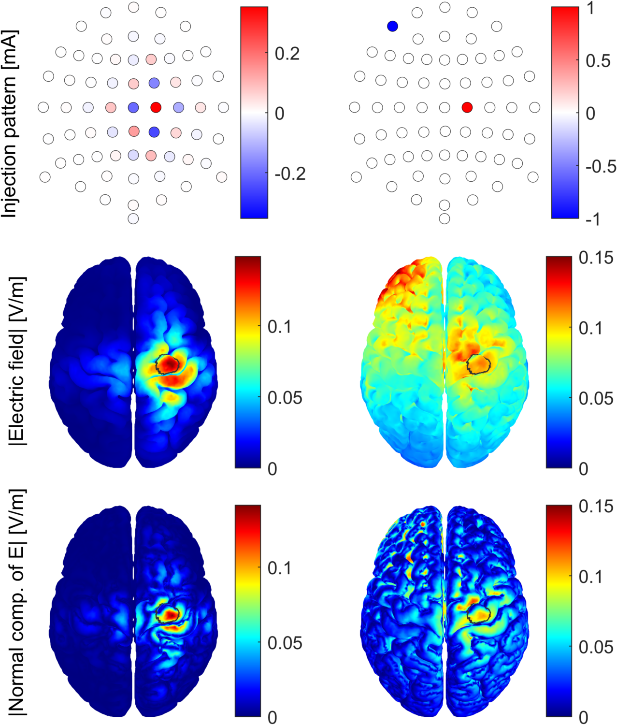


Figure S1: WLS closed-form solution from Eq. (5) scaled such that the total current injection budget is used (left column), and one-to-one reciprocity closed-form solution from Eq. (13) (right column). Note that the solution on the left column is identical to the iterative solution on the left columns of Fig. 1B, and that closed-form solution on the right column is equivalent to the iterative solution on the right column of Fig. 1B. As in Fig. 1B, the top row depicts the current injection patterns, the middle row depicts the modulus of the electric fields on the cortex and the bottom row depicts the intensity of the directional (normal-to-cortex) electric fields.


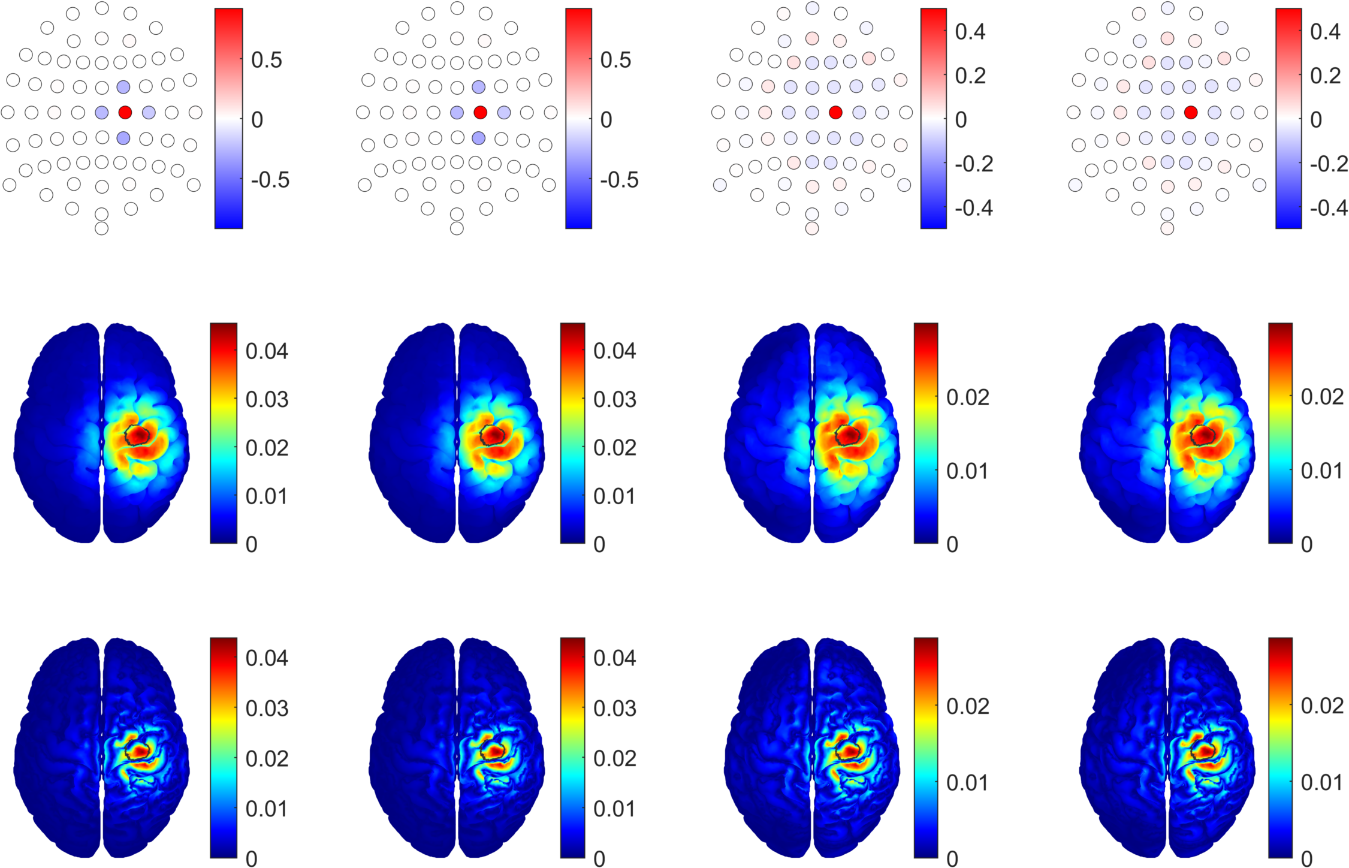


Figure S2: Left to right: Eqs. (7.a) and (7.b) are equivalent for a specific $\alpha'$ value (first two columns), and Eqs. (8.a) and (8.b) are equivalent for another value $\alpha^{''}$ (last two columns). In this example, $\alpha'\cong2.69\times{10}^{-8}$ results in Eqs. (7.a) and (7.b) to be equivalent and $\alpha''\cong1.82\times{10}^{-8}$ results in Eqs. (8.a) and (8.b) to be equivalent. For Eqs. (8.a) and (8.b), we set $\tilde{i}_{\max}=i_{max}/2$ and $\tilde{i}_{\min}=-i_{max}/20$, i.e. at least two sources and at least 20 sinks. The first column depicts ℓ_1_-constrained maximizing intensity solution to Eq. (7a) using the iterative SDPT3 solver. The second column depicts ℓ_1_-constrained WLS iterative optimization solution to Eq. (7b) also using SDPT3. The third column depicts total current (ℓ_1_ constraint) and current per electrode constrained maximizing intensity solution to Eq. (8a) using SDPT3. The fourth column depicts total current (ℓ_1_ constraint) and current per electrode constrained WLS iterative optimization solution to Eq. (8b) also using SDPT3. Top to bottom: the first row shows the injected current per electrode [mA], i.e., the optimal current injection pattern $\hat{i}$; the second row depicts the modulus of the electric field [V/m] on the cortex (i.e., the dose) where the $Ω_{\mathrm{ROI}}$ is circled in black; and the third row depicts the absolute value of the normal to cortex surface component of the electric field [V/m]. Note that in terms of the normal to cortex field, the stimulation focality is better.


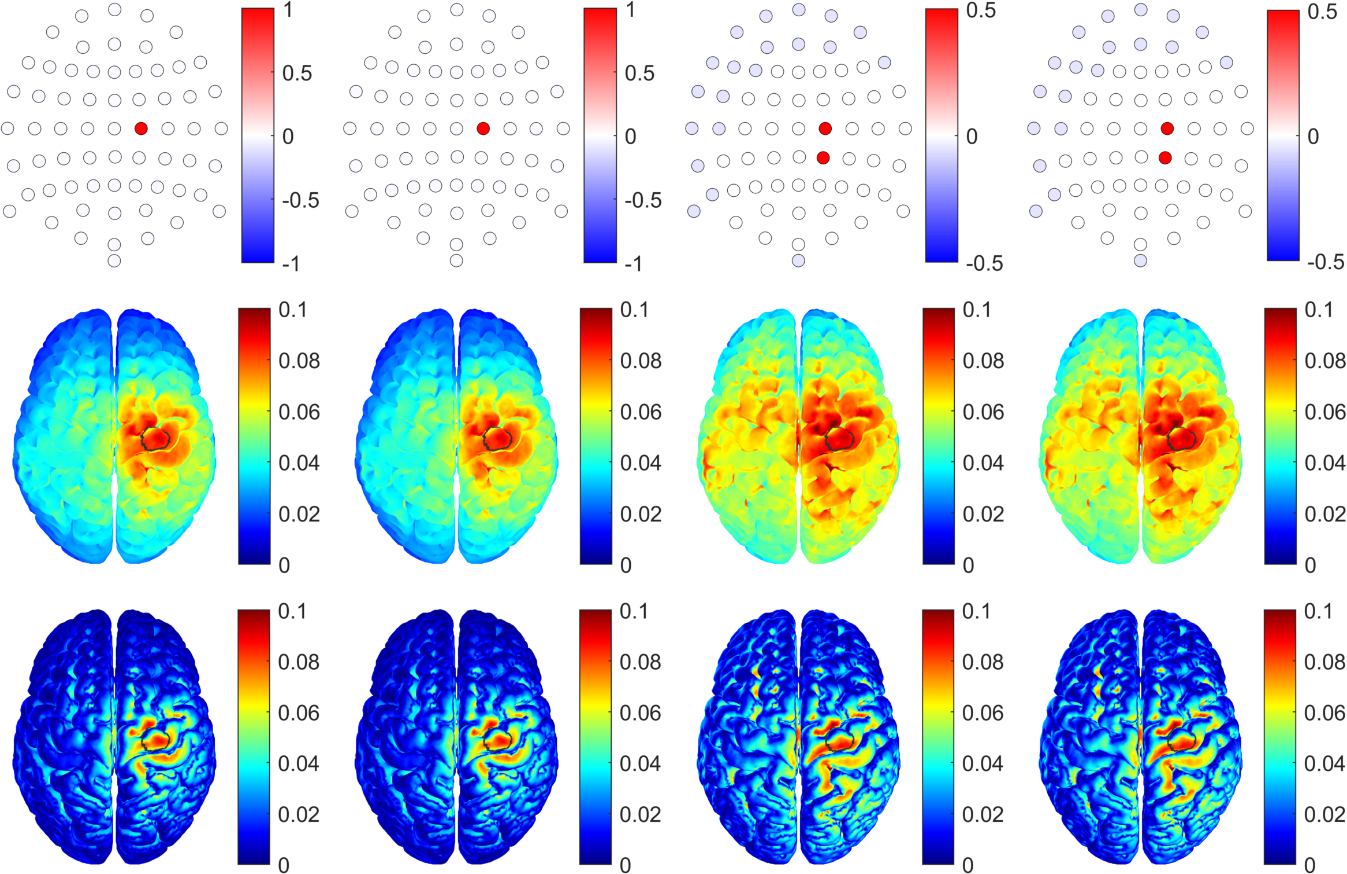


Figure S3: Equivalence between iterative solutions with large $\alpha$ (first and third columns) and reciprocity-based closed-form (second and fourth columns) solutions when considering limit current per electrode constraint in Eq. (3.iii) as $\tilde{i}_{\max}=i_{max}$ and $\tilde{i}_{\min}=-i_{max}/(L-1)$ (first and second columns); and $\tilde{i}_{\max}=i_{max}/2$ and $\tilde{i}_{\min}=-i_{max}/20$ (third and fourth columns). The first row shows the injected current per electrode [mA], i.e., the optimal current injection pattern $\hat{i}$; the second row depicts the electric field [V/m] on the cortex (i.e., the dose) where $Ω_{\mathrm{ROI}}$ is circled in black; and the third row depicts the absolute value of the normal to cortex surface component of the electric field [V/m]. Note that in terms of the normal to cortex field, the stimulation focality is better. As the first two columns are also used as an example of possible ways of reducing the strong unwanted electric field near the sink of the one-to-one reciprocity solution, the two focality metrics (Integral focality and Elementwise focality) were computed for it. All three focality metrics are indeed larger (better) than the one-to-one reciprocity solution (by 57% and 26%, respectively).


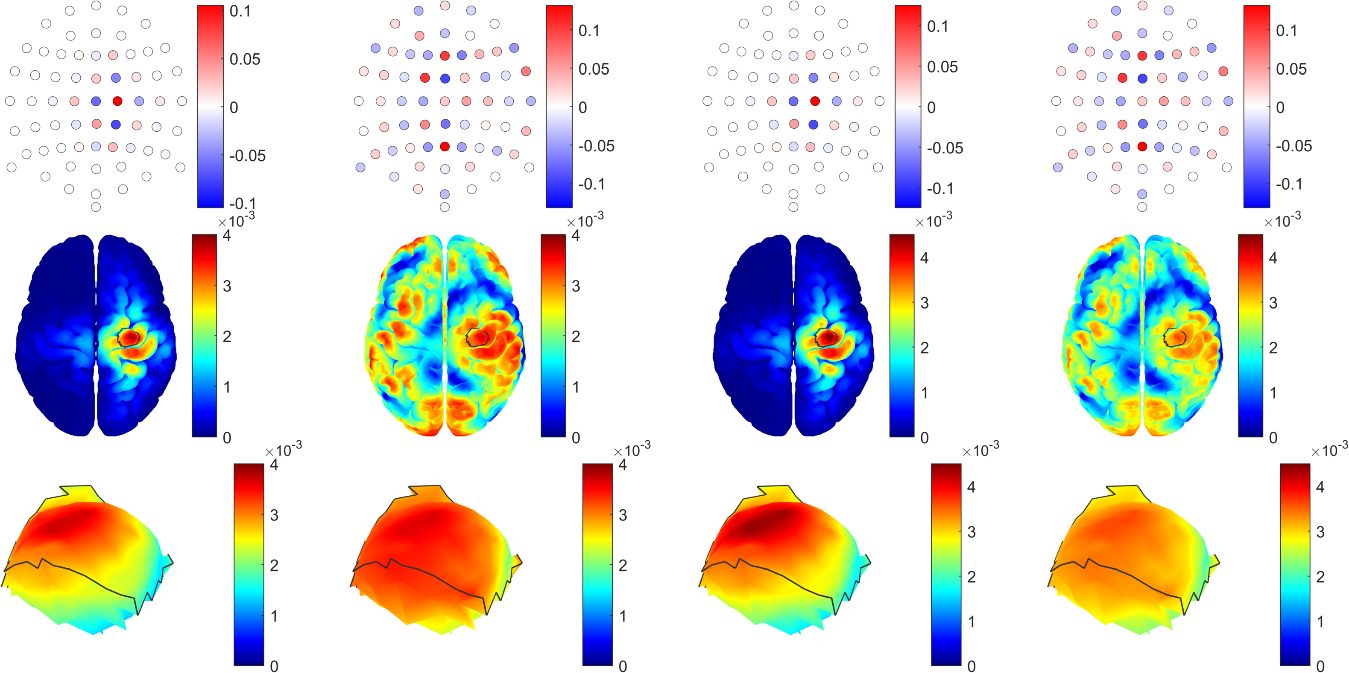


Figure S4: Comparison between the solutions when using the different $Ω_{non-ROI}$ constraints of Eqs. (3.i.a) and (3.i.b) for $\alpha$ values lower than critical point “a”. The first row shows the injected current per electrode [A], i.e., the optimal current injection pattern $\hat{\mathbf{i}}$; the second row depicts the electric field [V/m] on the cortex (i.e., the dose) where $Ω_{\mathrm{ROI}}$ is circled in black; and the third row depicts the absolute value of the normal component of the electric field only showing $Ω_{\mathrm{ROI}}$ from a side view [V/m]. The first two columns depict the solutions when the maximum $Ω_{non-ROI}$ absolute value of the electric field (or “secondary peak”) is the same (∼3.7 mV/m). Note that the peaks of the last row, first and second columns are the same, but the overall integral is smaller in the first column. The mean of the directional electric field at $Ω_{\mathrm{ROI}}$ is larger for the optimal pattern obtained with constraint of Eq. (3.i.b) (second column), ∼3 mV/m, than with constraint of Eq. (3.i.a) (first column), ∼2.5 mV/m. The last two columns depict the solutions when the mean of the directional electric field at $Ω_{\mathrm{ROI}}$ is the same (∼3 mV/m). This means that the integral of the directional electric fields at $Ω_{\mathrm{ROI}}$ is equivalent. The maximum $Ω_{non-ROI}$ absolute value of the electric field (or “secondary peak”) is smaller for the optimal pattern obtained with constraint of Eq. (3.i.b) (second row, fourth column), ∼3.7mV/m, than with constraint (3.i.a) (second row, third column), ∼4.4mV/m. In conclusion, the unintuitive pattern obtained when using constraint of Eq. (3.i.b) is better than any WLS scaled solution in terms of elementwise focality. This was expected from the theory, but as the pattern was not intuitive, an additional verification is provided in this example.
